# From the field to the pot: phenological, agronomic, and cookability traits of common beans (*Phaseolus vulgaris* L*.)* grown in contrasting climatic regions in Uganda

**DOI:** 10.3389/fpls.2026.1811268

**Published:** 2026-06-17

**Authors:** Ann Ritah Nanyonjo, Yuzhou Lan, Haftom Brhane, Mulatu Geleta, Stanley Tamusange Nkalubo, Dorothy Nakimbugwe, Ramune Kuktaite

**Affiliations:** 1 Department of Food Technology and Nutrition, Makerere University, Kampala, Uganda; 2 Department of Plant Breeding, Swedish University of Agricultural Sciences, Lomma, Sweden; 3 National Crops Resources Research Institute, Kampala, Uganda; 4 Rwebitaba Zonal Agricultural Research and Development Institute, Fortportal, Uganda

**Keywords:** cooking time, drought stress, legume breeding, phenotypic traits, seed color, yield

## Abstract

**Introduction:**

Common beans (*Phaseolus vulgaris* L.) phenotypic characteristics, cooking quality, and resilience to climate change are pivotal in making varietal choices of this crop in Uganda.

**Methods:**

This study evaluated 247 common bean genotypes across two climate-diverse regions, Mubuku (883 mm precipitation) and Namulonge (1300 mm precipitation), with 199 genotypes evaluated for phenological, agronomic, and cookability characteristics.

**Results:**

The mixed model analysis indicated significant differences in both genotype and genotype x environment effects for days to flowering and maturity, number of pods per plant, 100-seed weight, seeds per pod, and per plant (P < 0.001). The environment significantly influenced days to flowering and maturity (P < 0.001), while the genotype affected cooking time (P < 0.05), but not water absorption capacity. Significantly longer cooking time (75 minutes, P < 0.05) for red color seeds and shorter cooking time (62 minutes, P < 0.05) for white color seeds were found compared to the mean. The medium-seed-size genotypes had the shortest cooking time. Meanwhile, both the medium and large-seed-size genotypes exhibited high yields. Seven genotypes (NAROBEAN6, SMR48, SCR60, SCR45, SCN20, KND100, SCN744), which showed superior performance based on best linear unbiased predictor values for the traits studied, should be further tested across multiple climates and considered in common bean improvement programs.

**Discussion:**

The phenotypic trait, 100-seed weight, is important particularly when breeding for yield and cooking time. This study uniquely highlights the common bean genotypes that might be critically important in drought-stress environments and for the improvement of food security in Uganda and Africa at large.

## 1 Introduction

Common bean (*Phaseolus vulgaris* L.) has gained global importance due to its nutritional value, including proteins and micronutrients, as well as its affordability and ease of cultivation (Myers and Kmiecik, 2017; Rathna and Manickavasagan, 2020; Uebersax et al., 2023). In Africa, Uganda is the second-largest producer of common beans, with the crop ranking among the top ten commodities for food consumption (FAOSTAT, 2023). Common beans in Uganda by 2021 were grown on 962,000 hectares, producing 544,000 tones with an average yield of 0.7 million tones/Ha (UBOS, 2026). The choice of common bean varieties is usually made by farmers who also double as consumers and is driven by the varieties’ ability to fit soils, climatic conditions, and socio-economic demands (Weltzien and Christinck, 2017; Awio et al., 2018). Desired traits for common beans include phenological (days to maturity), agronomic (adaptation to environmental stress), yield, quality (grain color and size), and processing, *e.g.*, cooking time (Blair et al., 2006; Nkalubo et al., 2024; Mbiu et al., 2025). In addition, participatory varietal selection in Uganda has been driven by variety adaptation to both climatic variation and crop-growing stresses (Mukankusi et al., 2015; Awio et al., 2018).

The preference for early-maturing common bean varieties is popular among farmers in Africa (Wilkus et al., 2018; Nchanji et al., 2021; Babirye et al., 2024), due to the associated minimized impact of drought (Darkwa et al., 2016; Polania et al., 2016). However, there is still a need for common bean varieties that deliver satisfactory yields in diverse growing zones with fluctuating climates in Uganda.

Visual traits of common beans, such as grain size and color, are key characteristics for end-users (Nchanji et al., 2021; Swema and Mwinuka, 2021; Bogweh et al., 2023), substantially influencing the crop’s grain price and marketability. Generally, red-mottled, yellow, black, and white common beans dominate the market (Mukankusi et al., 2015; Babirye et al., 2024), and are also the most consumed types in Uganda (Asiimwe et al., 2024). Grain size and color are traits closely related to processing characteristics, e.g., cooking time, which is a key preference for common bean varieties among breeders and end-users (Berhanu et al., 2021; Cholo et al., 2023). Short cooking time of common beans was ranked among the most important intrinsic drivers for the choice of common bean varieties by end-users in Uganda (Asiimwe et al., 2024; Babirye et al., 2024).

Accordingly, varieties with relatively short cooking time (around 30 min.) save preparation time, labor, energy, and the environment, clearly indicating cooking time as an important breeding target for common beans. Normally, the cooking time of common beans ranges between 28 and 100 min. for soaked (Mughi et al., 2017) and 83–133 min. for the unsoaked types (Hedwig et al., 2017). Cooking time has largely been found to be controlled by the genotype (Katuuramu et al., 2020; Diaz et al., 2021; Amongi et al., 2023b; Kesiime et al., 2024). Moreover, the cooking behavior was found to be significantly influenced by several postharvest storage factors such as duration, temperature, and relative humidity, which cause the so-called ‘hard to cook’ defect in common beans (Wainaina et al., 2022; Perera et al., 2023; Kesiime et al., 2024). Cooking time has been related to seed coat color and thickness. Indeed, the white and yellow-seed types of common beans were among those with the shortest cooking time (around 29 min when soaked), and popular in South America and Africa (Amongi et al., 2021; Diaz et al., 2021; Beebe, 2012). However, knowledge gaps remain in understanding the genetic regulation of the fast-cooking trait, the interaction between the environment and genetic potential, and the biochemical mechanism behind the ‘hard-to-cook’ defect in common beans. Therefore, varieties that fulfil the demands of end-users, *e.g.*, high-yielding, cook fast, taste good, and are resistant to diseases and varying climates, are greatly desired in Uganda. Common bean varieties can be classified according to the 100-seed weight into small (< 25 g), medium (25–40 g), and large (> 40 g) according to their 100-seed weight (IBPGR, 1982; Blair et al., 2009), a categorization influencing the choice of varieties by end-users. Households with the highest variety adoption preferred large-seeded common beans in their seed stock (Wilkus et al., 2018), while farmers prioritized either medium and/or large-seeded varieties in Uganda (Kiwuka et al., 2012; Awio et al., 2018).

Although several studies on common bean in Uganda exist, including those focusing on the assessment of diversity (Kiwuka et al., 2012), traditional common bean varieties to manage bean fly (Ssekandi et al., 2016), and water use efficiency, and that examine grain yields, and economic benefits (Fatumah et al., 2021), there is still a lack of studies addressing the climate change impact on the phenotypic and quality traits. Therefore, an urgent need exist to breed for climate resilient, high yielding common bean varieties for the Ugandan consumer.

This study investigated a unique approach based on the phenological, agronomic, and cookability characteristics of a large germplasm collection of common beans grown in two climate-diverse regions in Wakiso and Kasese, including a drought-prone region, in Uganda. The novelty of the study lays in the inter-relation of the studied traits of common beans in a stress-growing environment, not investigated previously. The hypothesis was that diverse common bean genotypes show broad genetic variation in phenological, agronomic, and cookability traits across diverse climate environments, enabling the identification of widely adapted genotypes for further use in breeding programs. Thus, promising common bean genotypes can be identified according to their superior performances for the studied breeding traits across different years and growing environments in Uganda.

## 2 Materials and methods

### 2.1 Genetic material and field experiments

The genetic material used in this study comprised 23 common bean varieties released by the National Agricultural Research Organization (NARO) and 224 breeding lines from the International Centre for Tropical Agriculture (CIAT), both in Uganda. The genotypes were selected according to their diverse genetic backgrounds in terms of market class, and inherent traits, specifically, disease resistance, cooking quality, yield, iron, and zinc content (**Supplementary Table 1**). The common beans were grown in two locations. The first site was the NARO’s National Crops Resources Research Institute, situated in the Lake Victoria crescent region at Namulonge, Wakiso district (0°31′30′′ N, 32°36′54′′ E; 1160 meters above sea level, with an average annual rainfall of 1300 mm); further referred as ‘Namulonge’. The second site was Mubuku irrigation scheme in the Western Highlands region in Kasese district (0°15′41.00′′ N, 30° 7′27.00′′E; 1330 meters above sea level, with an average annual rainfall of 883 mm); further referred to as ‘Mubuku, a terminal drought-prone region’. The soils at Namulonge are classified as black red sandy loam, while those at Mubuku are generally of clay loam and sandy loam (SAVIMAXX, 2017; Tinkasimire et al., 2023; Wanyama et al., 2024).

The common bean collection, 247 genotypes, was planted in two growing seasons: from September 2023 to January 2024, and from April to August 2024. At Mubuku, planting was conducted one month later than at Namulonge to target the terminal drought stress that occurs in the Kasese district. During the growing seasons, Mubuku experienced more water stress compared to Namulonge (**Supplementary Figure 1**). Similarly, Namulonge had lower temperatures ranging between 27-29 °C in season one and 28-29 °C in season two, Kasese had ranges 28-31 °C in season one and 30-32 °C in season two (**Supplementary Figure 1**).

The experiments were laid out in an alpha lattice design with three replicates of each genotype in both seasons and locations. Genotypes were planted in two 1.5-m rows per plot, with a spacing of 10 cm between plants and 50 cm between rows. In season one, each of three replicates consisted of 19 blocks with 13 plots (genotype) per block. In season two, each of the three replicates comprised 15 blocks, and 13 plots per block. Agronomic practices, such as weeding and application of fungicides and pesticides, were carried out whenever symptoms were observed. It is noteworthy that in season one, 48 genotypes succumbed to sclerotium root rot disease and were lost in the field in all locations before data were collected; thus, 199 were used for data collection, and the lost accessions were considered as missing plots in the analysis. Accordingly, 195 genotypes were planted in season two to balance the alpha lattice design, considering genotypes that had enough seeds to be planted in both locations.

### 2.2 Data collection on the phenological, agronomic, and cookability traits

The phenological, agronomic, and cookability traits evaluated in this study are shown in **Table 1**. The phenological traits included: days to 50% flowering (DF) and days to physiological maturity (DPM). Agronomic traits comprised: number of pods per plant (Pod_P), number of seeds per pod (S_pod), number of seeds per plant (Seed_P), 100-seed weight (Wt_100S), plot yield (PYLD), seed coat primary color, and seed shape. Cookability traits involved: cooking time (CT) and water absorption capacity (WAC). We refer to the seed coat primary color as ‘seed color’ and days to 50% flowering as ‘days to flowering’ in the rest of the text.

**Table 1:** Description of the traits of common beans targeted in this study.

Trait name	Description	Type of trait
Days to 50% flowering (DF)	Number of days from planting to the day when 50% of the plants have flowered (IBPGR, 1982).	Phenological
Days to physiological maturity (DPM)	Number of days from planting to the day when the first pod begins to discolor in 50% of the plants (IBPGR, 1982).	Phenological
Number of pods per plant (Pod_P)	The average number of pods from 10 randomly selected plants (IBPGR, 1982).	Agronomic
Number of seeds per pod (S_pod)	The average number of seeds counted from 10 randomly selected pods (IBPGR, 1982).	Agronomic
Number of seeds per plant (Seed_p)	The average number of seeds counted from 10 randomly selected plants (IBPGR, 1982).	Agronomic
Seed color	1- white, 2- cream-beige, 3- yellow, 4-brown-maroon, 5- pink, 6- red, 7- purple, 8- black, 9- khaki (IBPGR, 1982).	Agronomic
100-Seed weight (Wt_100S)	Small: < 25 g, medium: 25–40 g, large: > 40 g (IBPGR, 1982)	Agronomic
Yield (kg/ha) (PYLD)	Weight of bulk seeds from each plot in g and converted to kg/ha (Amongi et al., 2023b)	Agronomic
Cooking time (min) (CT)	Time taken for 80% of the pins of the Mattson machine to penetrate the seed (Wang and Daun, 2005)	Cookability
Water absorption capacity (WAC, %)	$\frac{\left(W2\;(g)-W1(g)\right)}{W1\;(g)}*100$ (Diaz et al., 2021)	Cookability

#### 2.2.1 Agronomic traits

For each plot of the 199 genotypes across all the replicates, ten plants were randomly selected at harvest and individually used to collect data on the yield components, including the number of pods per plant (Pod_P), number of seeds per pod (S_pod), and number of seeds per plant (Seed_P) (**Table 1**). The mean values for these traits were then calculated from the ten plants. All common bean seeds harvested from each plot were bulked in paper bags, sun-dried, sorted to remove debris, and weighed in grams to obtain plot yield (kg/ha). Thereafter, 100 seeds were randomly selected from the bulk and weighed to determine the 100-seed weight (Wt_100S). The 100 seeds were subsequently used to determine the seed color and shape (**Table 1**).

#### 2.2.2 Cookability traits

##### 2.2.2.1 Water absorption capacity

Water absorption capacity (WAC) and cooking time were measured for 199 common bean genotypes, which were grown at Mubuku and Namulonge in 2023. Accordingly, for WAC, twenty-eight seeds of each genotype were weighed (W_1_, g), soaked in distilled water for 18 hours at room temperature, drained of water, and weighed (W_2_, g) to determine WAC according to the formula by Diaz et al. (2021), $\frac{\left(W2\;(g)-W1(g)\right)}{W1\;(g)}*100\%$. where W2=soaked seed weight, g; W1=unsoaked seed weight, g.

##### 2.2.2.2 Cooking time

Cooking time was determined using the automated Mattson cooker (Customized Machining and Hydraulics Co., Winnipeg, Canada, modified at the International Centre for Tropical Agriculture) according to the method by Wang and Daun (2005) with some modifications. The Mattson cooker consisted of a plate with 25 wells used to hold individual common bean seeds, together with a plunger, which was calibrated with a specific weight (90 g), which terminates in a stainless steel 2 mm diameter probe. Twenty eight common bean seeds of each genotype from each replicate, and location were randomly selected, soaked in water at room temperature for 18 h. Twenty-five soaked seeds were randomly selected and placed horizontally in the wells of the Mattson cooker, with each well having a single seed so that the tip of each plunger rested on top of the seed. The extra seeds were discarded. The Mattson cooker was placed in a two liter beaker containing 1800 ml of boiling distilled water at >98°C, which was maintained during the experiment. During boiling when a seed softened, the probe penetrated the seed and dropped the plunger, and cooking time was recorded as the time when the probe had penetrated 80% of the seeds (Wang and Daun, 2005).

### 2.3 Data analysis and statistical model description

Data was collected over two seasons, with 199 genotypes evaluated in Season 1 and 195 genotypes in Season 2. Descriptive statistics for phenological (DF and DPM), agronomic (Pod_P, Seed_P, S:pod, Wt_100S, PYLD), and cookability (CT, WAC) traits of common bean were computed using the R software environment. As the dataset included different numbers of genotypes across seasons, resulting in an unbalanced design, missing observations were coded as “NA” to ensure compatibility with the META-R version 6 statistical software (Alvarado et al., 2020), which was used to perform a combined analysis of variance (ANOVA). The ANOVA was conducted with a mixed linear model using lmer package in R software (Bates, 2015) run within META-R. All variables were treated as random effects to estimate variance components. The META-R statistical software implements the mixed linear model using the lmer function from the lme4 package of R software. The lmer function uses Restricted Maximum Likelihood (REML) to estimate variance components and calculate best linear unbiased predictors (Alvarado et al., 2020). The use of REML within the lme4 package was chosen because this model provides unbiased estimates of variance components in unbalanced designs (Bates, 2015; Maestrini et al., 2024). The model run within the lmer function was limited to handle many factors, and therefore, seasonal effects were excluded. The genotype, environment and genotype by environment (G×E) interaction effects on the total phenotypic variance of each trait were then determined using the model below.


$$y_{ijkl}=\mu +G_{i}+{\text{E}}_{j}+{\text{GE}}_{ij}+{\text{I}}_{k(j)}+\;\delta_{l(ijk)}+\epsilon_{ijkl}$$

i=1, …, g; j=1,…,e;  k=1,…, r; l=1,…,c 

where $y_{ijkl}$ is the response variable (phenological, agronomic, and cookability), μ is the overall mean, $G_{i}$ is the effect of the ${\text{i}}^{th}$ genotype and g is the total number of genotypes, ${\text{E}}_{j}$ is the effect of the ${\text{j}}^{th}$ environment and e is the total number of environments, ${\text{GE}}_{ij}$ is the effect of the genotype by environment interaction, ${\text{I}}_{k(j)}$ is the effect of the $k^{th}$ replication nested in the ${\text{j}}^{th}$ environment and r is the total number of replications, $\;\delta_{m(ijl)}$ is the effect of the $l^{th}$ incomplete block nested in the $k^{th}$ replication and ${\text{j}}^{th}$ environment and c is the total number of incomplete blocks, $\epsilon_{ijkl}\;$is the error associated to the experimental unit (i.e. ${\text{ijkl}}^{th}$ plot). Then, ${\text{G}}_{i}∼\;N(0,\sigma_{G}^{2}),{\text{ E}}_{j}∼\;N(0,\sigma_{E}^{2}),$ ${\text{GE}}_{ij}∼\;N(0,\sigma_{GE}^{2})$, ${\text{I}}_{\text{k}(j)}\;∼\;N(0,\sigma_{I}^{2}),$ $\;\delta_{l(ijk)}∼\;N(0,\sigma_{\delta }^{2})$, $\epsilon_{ijkl}∼\;N(0,\sigma_{\epsilon }^{2})$, and covariances among random effects terms were zero.

Subsequently, best linear unbiased predictions (BLUPs) and heritability estimate for the traits studied were obtained. Broad-sense heritability (H^2^) was calculated in META-R according to the formula (Alvarado et al., 2020):


$$H_{2}=\frac{{\text{σ}}^{2}\text{g}}{\begin{array}{c} {\text{σ}}^{2}g+\frac{{\text{σ}}^{2}ge}{nE}+\frac{{\text{σ}}^{2}e}{\left(nE\times nR\right)} \end{array}}$$

where, σ^2^g is the genotypic variance, σ^2^ge is the genotype-by-environment variance, σ^2^e is the environmental variance, nE is the number of environments, and nR is the number of replicates.

The BLUPs used to: 1) estimate correlations among traits using Pearson’s correlation implemented in the *ggplot2* package in R software (https://ggplot2.tidyverse.org/); 2) group genotypes using hierarchical clustering using the *pheatmap* package in R (https://github.com/raivokolde/pheatmap); 3) identify genotypes with superior performance.

Superior genotype selections were made based on BLUP values with cluster analysis. In the clustering analysis, the complete linkage hierarchical clustering method was used to define the distances between clusters. Four trait categories were established for genotypic ranking: phenological traits (DF and DPM), yield components (Pod_P, Seed_P, S_pod), plot yield (PYLD), and cookability (CT). All the traits were ranked, and then the rank numbers of the traits belonging to the same trait category were averaged to represent the rank of each genotype’s performance in phenology, yield components, plot yield, and cookability separately. Subsequently, the rank values of those four trait categories were averaged into one to represent the overall rank of each genotype, and this value was used as the final criterion to select superior genotypes with small overall rank values. The ranking outcome for the 199 genotypes is listed in **Supplementary Table 2**. Principal component analysis (PCA) was conducted using the R package ‘ggfortify’ to display the distribution of genotypes across clusters and to visualize the trait-genotype relationships (**Supplementary Figure 2**). In addition, analysis of means of cooking time and plot yield was done using Minitab software (version 21.4.3). The *ggplot2* package in R software was used to illustrate the physical variation of the genotypes in terms of seed color, size, and shape. A descriptive analysis for the released varieties and breeding lines to evaluate their separate performance was conducted using the software Excel.

## 3 Results

### 3.1 Seed color, shape, and size

**Figure 1:**
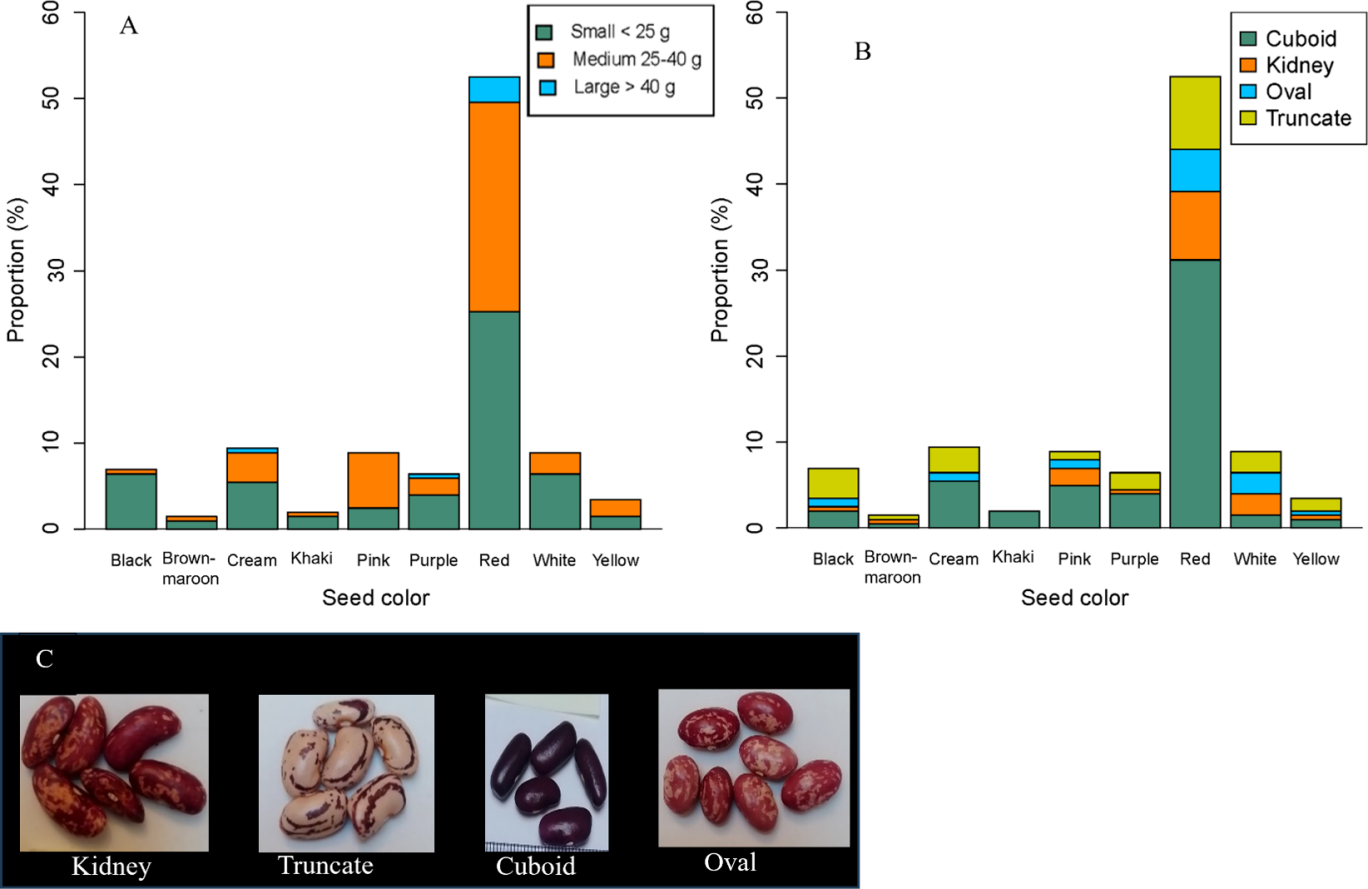
Distribution of common bean seed morphology, categorized by **(A)** 100-seed weight, **(B)** seed shape, and **(C)** visual appearance of common beans with different seed shapes and colors.

The common bean genotypes used in this study varied in seed color, shape, and size, as shown in **Figure 1**. A clear dominance of red colored seeds of small (25.25%) or medium (24.26%) sizes was observed in the material (**Figure 1A**). White, cream, pink, and black colored seeds with small, medium, or large sizes were also found in smaller amounts. In terms of shapes, cuboid, truncated, and kidney types were, respectively, observed in larger proportions as compared to oval shape seeds (**Figure 1B**).

### 3.2 Variation in phenological, agronomic, and cookability traits of common beans grown in the climate-diverse regions

Heritability was highest for Wt_100S, with a value of 0.89 (**Table 2**). This was followed by DF, DPM, and Pod_P with respective heritability values of 0.88, 0.79, and 0.78 (**Table 2**). Cooking quality had the lowest heritability, specifically 0.39 for CT and 0.23 for WAC (**Table 2**). Genotypes differed significantly for most traits studied at P < 0.001, except for CT, which was significant at P < 0.05, and WAC, which did not differ significantly, indicating diversity within the genotype collection (**Table 2**). The breeding lines had slightly higher values for phenological traits, yield components, and cooking time (**Supplementary Table 3**).

**Table 2:** Analysis of variance of components, genotype (G), environment (E), and their interaction (G x E) of the evaluated agronomic, phenological, and cookability traits of common beans grown at Mubuku and Namulonge.

Components	DF	DPM	Pod_P	Seed_p	S_pod	Wt_100S (g)	PYLD (kg/ha)	CT (min)	WAC (%)
Heritability	0.88	0.79	0.78	0.81	0.76	0.89	0.57	0.39	0.23
Genotype (G)	5.8***	8.28***	4.88***	127.52***	0.33***	34.76***	97792.5***	73.66*	85.03
Environment (E)	4.14***	14.13***	1.88*	32.88*	0.1*	7.64***	55236.4**	217.55*	99.07*
G x E	1.64***	2.62***	1.85***	47.02***	0.16***	7.38***	164264***	37.25	24.87
Residual	4.87	18.12	11.29	214.32	0.81	27.85	403409	585.03	1618.32
Grand mean	38.52	69.08	8.83	32.16	3.89	27.25	1227.47	72.56	209.06
CV	5.73	6.16	38.03	45.52	23.17	19.36	51.74	33.34	19.24
n Replicates	3	3	3	3	3	3	3	3	3
n Environments	4	4	4	4	4	4	4	2	2

DF, Days to flowering; DPM, Days to physiological maturity; Pod_P, pods per plant; Seed_P, Seeds per plant; S_pod, Seeds per pod; Wt_100S, 100-seed weight (g); PYLD, Plot yield (kg/ha); CT, Cooking time (min); WAC, Water absorption capacity (%); P Values (< 0.001= ***, < 0.01 = **, < 0.05 = *)

**Figure 2:**
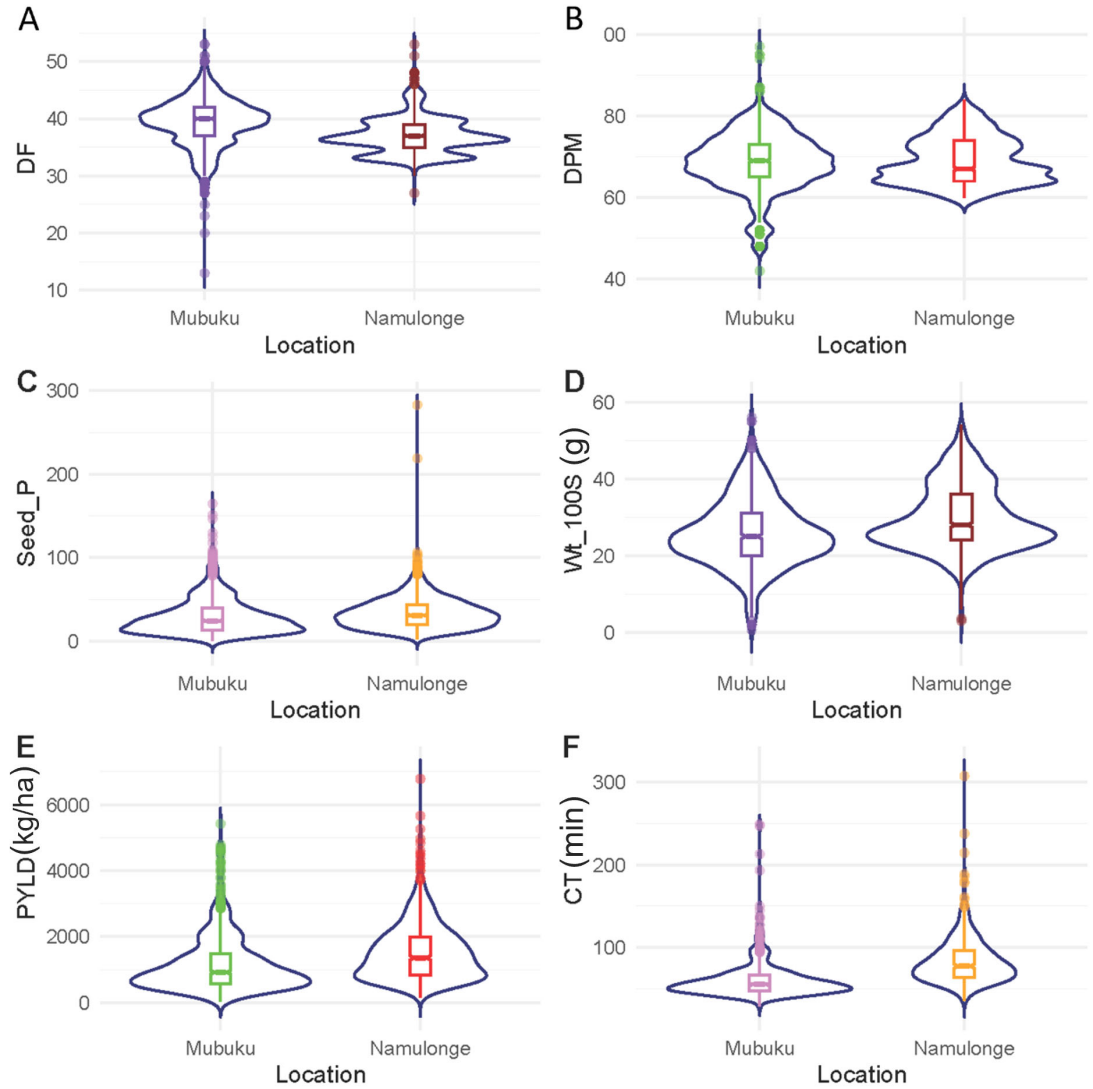
Distribution of genotype performance in Mubuku and Namulonge evaluated by, **(A)** days to flowering (DF), **(B)** days to physiological maturity (DPM), **(C)** seeds per plant (Seed_P), **(D)** 100-seed weight (g) (Wt_100S), **(E)** Plot yield (kg/ha) (PYLD), and **(F)** Cooking time (min) (CT).

The environment had significant effects on DF, DPM, and Wt_100s at P < 0.001, on PYLD at P < 0.01, and on all the other traits at P < 0.05 (**Table 2**). Genotype-by-environment interactions were significant (P < 0.001) for all the traits except CT and WAC (**Table 2**). Genotypes established at Namulonge had shorter days to flowering (DF) and days to physiological maturity (DPM) as compared to Mubuku (**Figure 2**). The genotypes in Namulonge also exhibited higher seeds per pod (Seed_P), larger 100-seed weight (Wt_100S) and higher plot yield (PYLD) as compared to Mubuku (**Figure 2**). The seeds harvested from the Mubuku field trial had shorter cooking time (CT) than those from Namulonge (**Figure 2**).

### 3.3 Phenological, agronomic, and cookability performance of the genotypes

**Figure 3:**
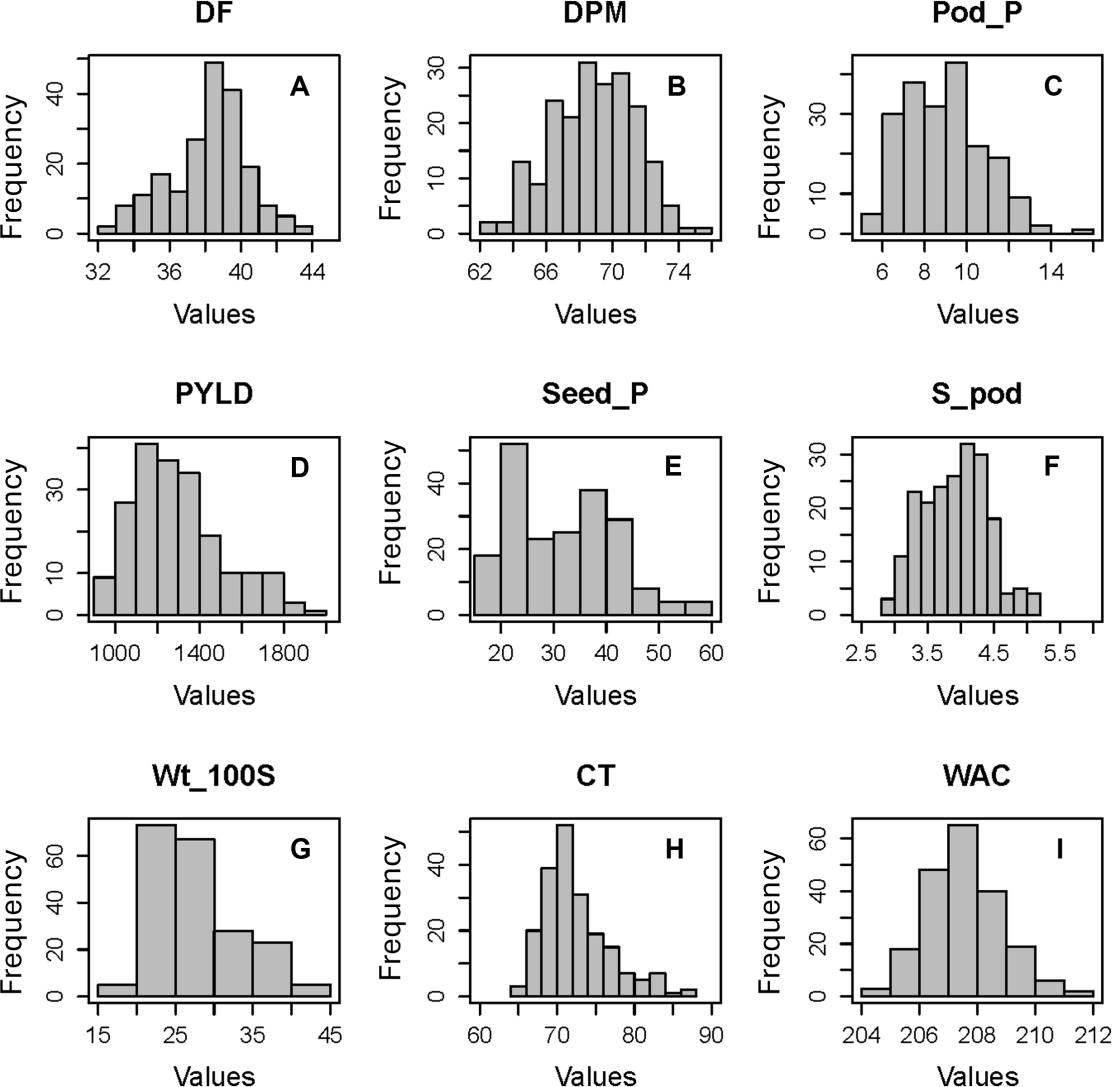
Histograms illustrating the frequency distributions of best linear unbiased predictors (BLUPs) for, **(A)** days to flowering, **(B)** days to physiological maturity, **(C)** pods per plant, **(D)** seed yield, **(E)** seeds per plant, **(F)** seeds per pod, **(G)** 100-seed weight (g), **(H)** Cooking time (min), and **(I)** water absorption capacity (%).

The performance of genotypes showed a bimodal pattern for DF, Pod_P, Seed_P, and Wt_100S, which may indicate the influence of major genes (**Figures 3A, C, E, G**), while DPM, S_pod, CT, and WAC displayed a normal distribution, suggesting polygenic control (**Figures 3B, F, H, I**). The values for the evaluated traits varied as follows: 32 to 44 for days to flowering, 62 to 76 for days to physiological maturity, 5 to 16 for pods per plant, 800 to 1900 kg/ha for yield,15 to 65 for seeds per plant, 2 to 6 for seeds per pod, 15 to 45 g for 100-seed weight, 63 to 87 minutes for cooking time, and 204 to 212% for WAC (**Figures 3A–I**).

**Figure 4:**
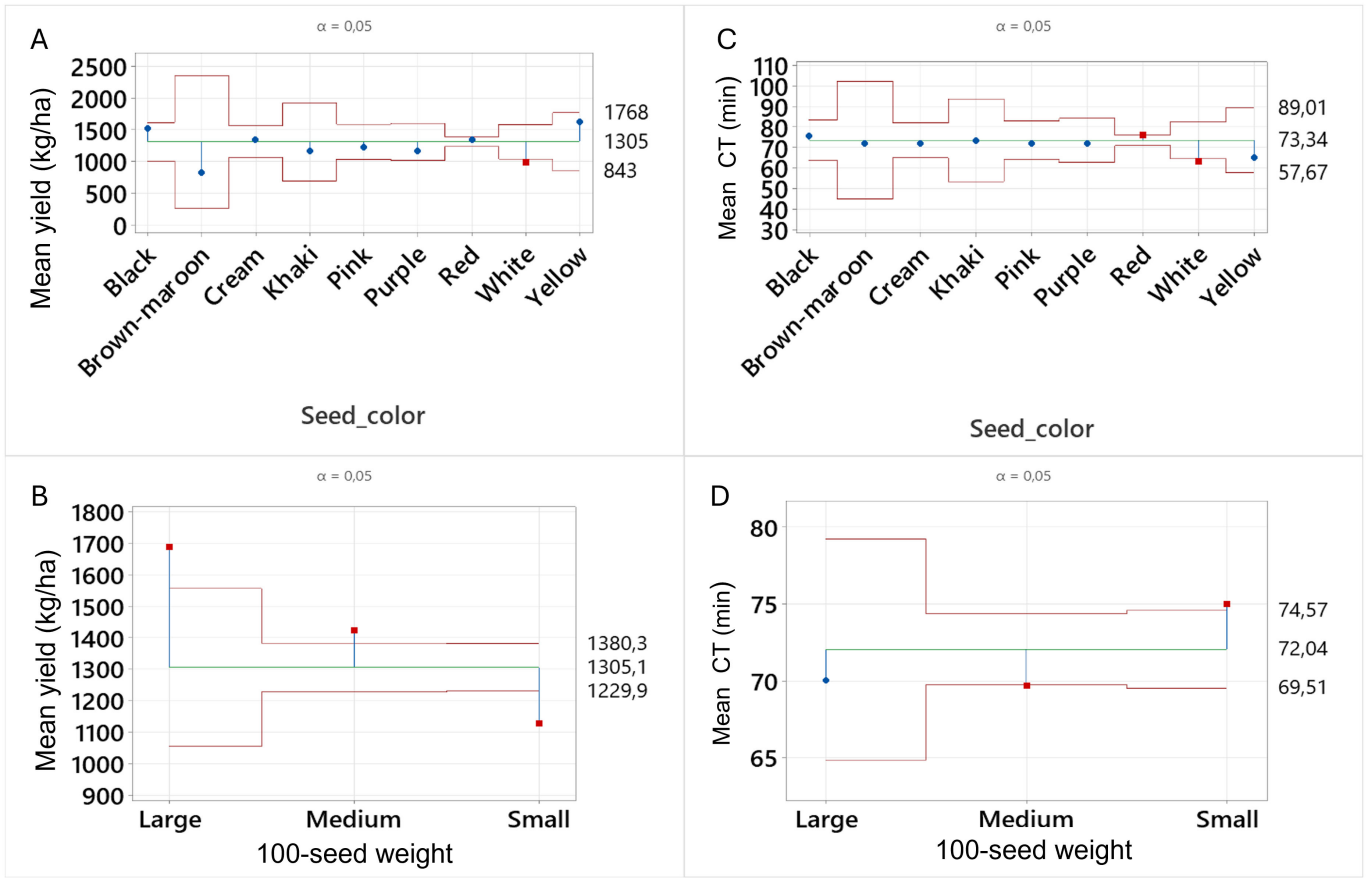
Analysis of means (± standard deviation) of genotypes categorized by seed color and 100-seed weight for, mean yield **(A, B)** and mean cooking time **(C, D)**. Blue markers indicate that the means are not significantly different from the overall mean at α = 0.05, while red markers indicate that the means are significantly different from the overall mean. CT, cooking time (min).

Variation in yield and cooking time for common bean genotypes grouped by seed color and size is shown in **Figure 4**. Genotypes with white seeds had significantly lower yield and cooking time compared to genotypes with red seeds (**Figures 4A, C**). Red seeds had significantly higher cooking time compared to seeds of other colors (**Figure 4C**). Furthermore, genotypes within various 100-seed weight categories exhibited significant differences for yield and cooking time (**Figure 4**). Genotypes with large seeds had significantly higher yield (**Figure 4B**), while those with small seeds had significantly lower yield (**Figure 4B**). Meanwhile, the medium-sized genotypes had significantly lower cooking time (**Figure 4D**), while the small-sized ones had significantly higher cooking time (**Figure 4D**).

### 3.4 Correlation between phenological, agronomic, and cookability

**Figure 5:**
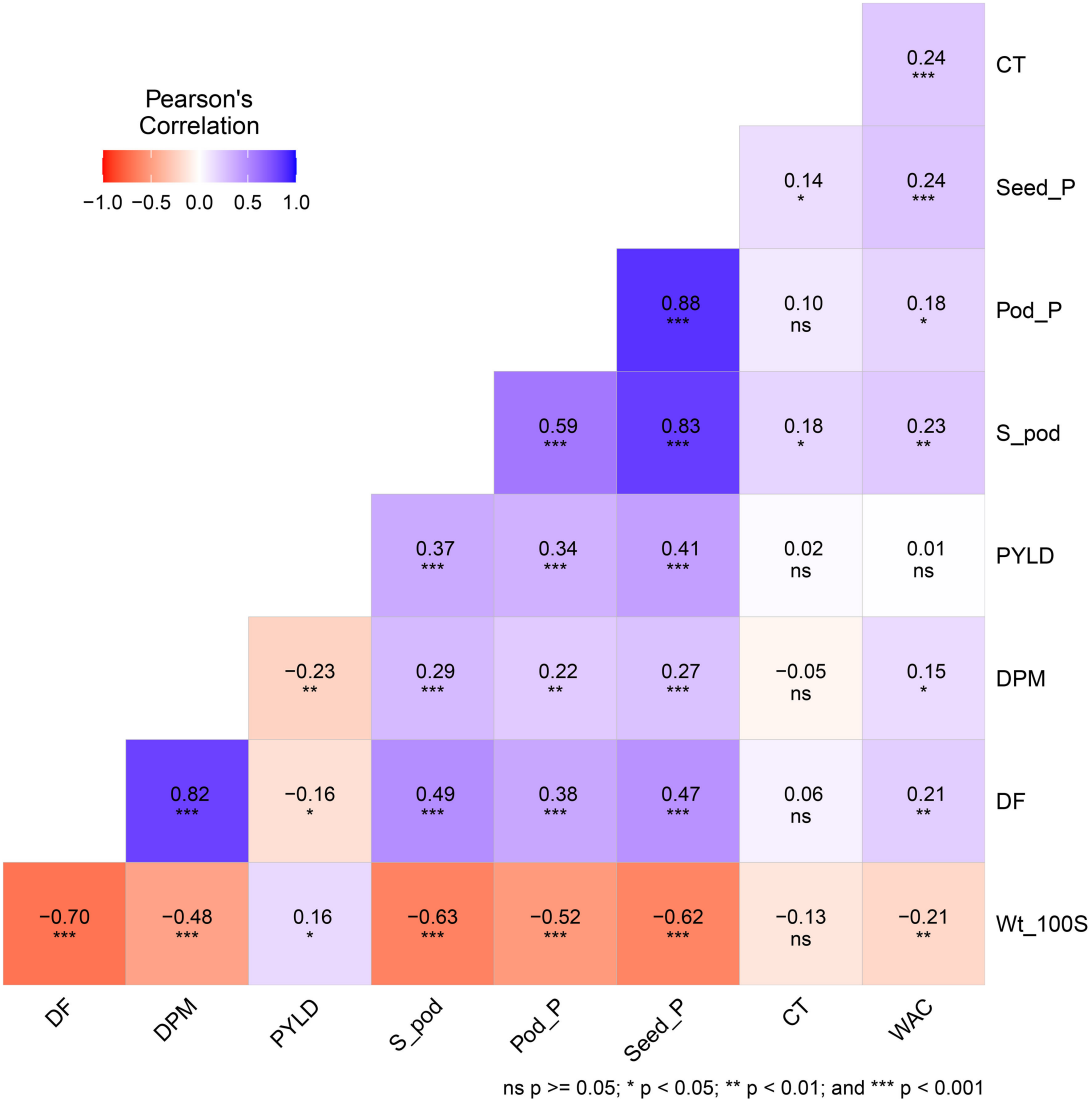
Correlation between phenological, agronomic, and cookability traits evaluated in this study based on Best Linear Unbiased Predictors (BLUPs). The traits include days to 50% flowering (DF), days to physiological maturity (DPM), Plot yield (kg/ha) (PYLD), seeds per pod (S_pod), pods per plant (Pod_P), seeds per plant (Seed_p), 100-seed weight (g) (Wt_100S), Cooking time (min) (CT), and Water absorption capacity (%) (WAC). ns p>=0.05; *p <0.05; **p <0.01; ***p <0.001

Significant positive correlations were observed among several agronomic and phenological traits. The strongest correlation was detected between seeds per plant and pods per plant (r = 0.88, P < 0.001), followed by seeds per plant and seeds per pod (r = 0.83, P < 0.001), and days to flowering and days to physiological maturity (r = 0.82, P < 0.001) (**Figure 5**). Days to flowering showed a moderate positive correlation with yield components, including seeds per pod (r = 0.49, P < 0.001) and seeds per plant (r = 0.47, P < 0.001) (**Figure 5**). Yield was moderately and positively correlated with seeds per plant (r = 0.41, P < 0.001) (**Figure 5**). In contrast, 100-seed weight exhibited strong negative correlations with days to flowering (r = −0.70), seeds per pod (r = −0.63), seeds per plant (r = −0.62), and pods per plant (r = −0.52), all significant at P < 0.001 (**Figure 5**).

### 3.5 Cluster analysis

**Figure 6:**
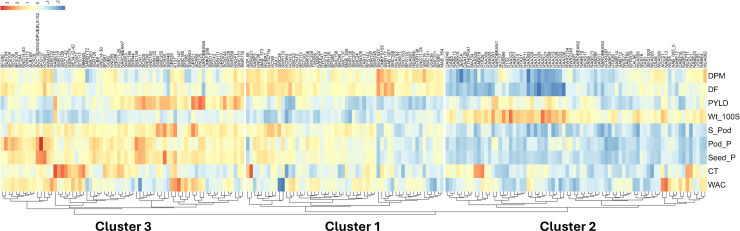
Hierarchical clustering of genotypes based on best linear unbiased predictors (BLUPs) for phenological, agronomic, and cookability traits: days to flowering (DF), and days to physiological maturity (DPM), plot yield (kg/ha) (PYLD), 100-seed weight (g) (Wt_100S), seeds per pod (S_Pod), pods per plant (Pod_P), seeds per plant (Seed_P), Cooking time (min) (CT), and Water absorption capacity (%) (WAC).

Cluster analysis based on agronomic, phenological, and cookability traits grouped the genotypes into three distinct clusters, each characterized by unique trait profiles (**Figure 6**). Cluster 1 mainly comprised genotypes with higher days to flowering and physiological maturity, and lower seed yield (**Figure 6**). Cluster 2 contained genotypes with a larger 100-seed weight, lower days to flowering, and days to physiological maturity (**Figure 6**). Cluster 3 included genotypes with higher seed yield and yield components. This cluster contained the largest number of genotypes with high cooking time and water absorption. Seven genotypes (NAROBEAN6, SMR48, SCR60, SCR45, SCN20, KND100, SCN744) in this cluster showed superior performance in relation to the mean across all evaluated traits (**Table 3**). The PCA result confirmed the clear separation of the three clusters among the four trait categories, with the seven highlighted genotypes contained in cluster 3 showing high values in plot PYLD and yield components, and relatively low values in DF and DPM (**Supplementary Figure 2**), confirming the validity of BLUP-ranking based genotype selection. Overall, the performance of genotypes evaluated in this study for different traits is highlighted in **Supplementary Table 2**.

**Table 3:** Trait BLUP values of the selected superior common bean genotypes based on the ranking of four trait categories i.e., phenological traits, yield components, grain yield and cookability.

Genotype	DF	DPM	Pod_P	Seed_P	S_pod	PYLD	CT	Wt_100S	Picture of seeds
NAROBEAN6	35.00	66.67	11.72	42.86	4.24	1808.85	68.84	24.48	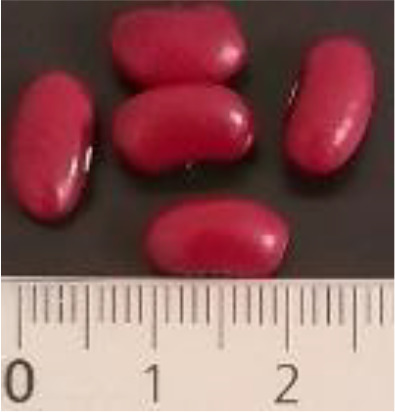
SMR48	35.66	65.60	9.25	41.60	4.55	1663.18	68.88	25.86	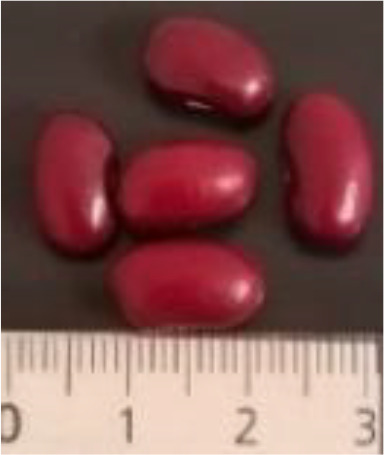
SCR60	38.28	67.99	9.07	45.80	4.83	1727.02	67.55	25.04	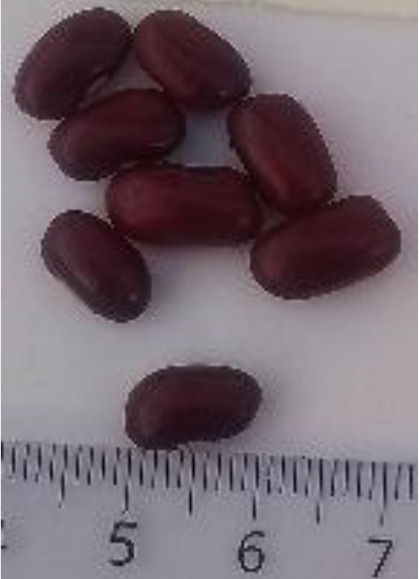
SCR45	37.14	66.40	10.81	60.11	5.15	1673.79	71.34	23.45	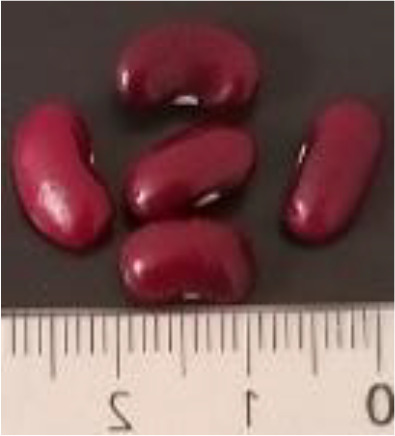
SCN20	37.90	68.58	12.68	51.21	4.43	1771.74	70.45	26.24	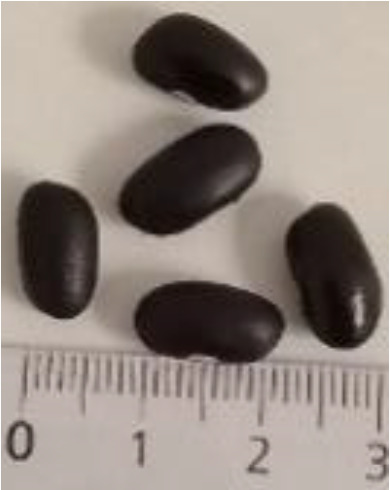
KND100	38.46	67.21	11.36	40.61	4.34	1657.18	69.52	25.65	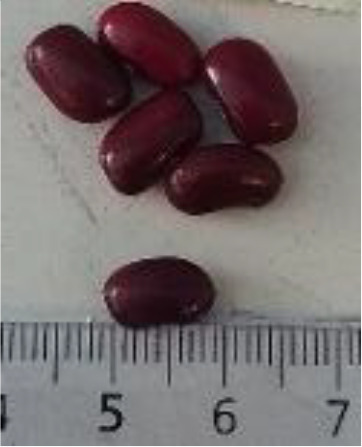
SCN744	37.74	70.44	9.40	37.21	4.44	1737.36	68.70	26.03	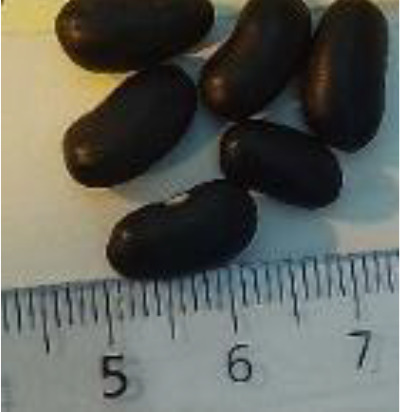

DF, Days to flower; DPM, Days to physiological maturity; Pod_P, pods per plant; Seed_P, Seeds per plant; S_pod, Seeds per pod; PYLD, Yield; CT, Cooking time; Wt_100S, 100-seed weight. The superior performing genotypes were selected based on those that had a superior BLUP value than the average BLUP for each trait across all the traits.

## 4 Discussion

Phenotypic characteristics of common bean seeds, specifically 100-seed weight, shape, and color, are key attributes determining the crop’s market classes and are of economic value (Assefa et al., 2019; Uebersax et al., 2022; Plestenjak et al., 2024; Mbiu et al., 2025). Similarly, seed color was identified as a key factor that influenced common bean varietal market popularity and price (Swema and Mwinuka, 2021; Bogweh et al., 2023). In this study, sorting the common bean genotypes by seed color revealed a predominance of red-seeded genotypes, which accounted for over 50% of those studied. This dominance reflects a strong end-user preference and market demand for red mottled beans in Uganda, which are well known for their profitability and availability (Asiimwe et al., 2024; Babirye et al., 2024). Similar preferences have been reported elsewhere, for instance, in Cameroon, where red beans were favored for being affordable, high-yielding, early-maturing, and tasty (Bogweh et al., 2023). Differences in red color hue, ranging from light to dark, are known to influence varietal acceptability and market class differentiation, particularly in Latin American countries (Voysest and Dessert, 1991; Mbiu et al., 2025).

In the present study, white seed-colored bean genotypes exhibited relatively shorter cooking times than their counterparts, consistent with earlier findings (Katuuramu et al., 2020; Diaz et al., 2021). The white seed coat color of common beans has been reported to be associated with low tannin concentrations, which contribute to shorter cooking times (Díaz et al., 2010; Jeffery et al., 2025). Since firewood and charcoal are the primary sources of energy used to cook common beans in Uganda (Kajumba et al., 2022; Asiimwe et al., 2024). Yet, these are environmentally unsafe, expensive, and laborious to obtain; there is a strong demand for breeding genotypes with short cooking times. Indeed, such varieties will contribute to lower household energy costs, labor burdens, and a safer environment. Additionally, common beans with short cooking times are likely to be more attractive to consumers in contexts where this trait strongly influences the market position of the variety.

It is also important to note that market preference of common bean varieties often favors medium- to large-seeded types (Kiwuka et al., 2012; Awio et al., 2018). This preference is supported by our results, which showed that medium and larger-seeded genotypes produced higher yields and exhibited shorter cooking times than small-seeded genotypes. These findings further suggest that 100-seed weight in common beans is closely associated with end-user trait preferences and, consequently, market popularity, highlighting the need to incorporate this trait into breeding objectives.

The highly significant genotypic variance observed for most traits targeted in this study, along with the relatively high heritability estimates (**Table 2**), indicate that genetic differences among genotypes had a substantial contribution to the observed variation, implying good prospects for selection. This was evident especially for seeds per plant (heritability 0.81), 100-seed weight (heritability 0.89), days to flowering (heritability 0.88), days to physiological maturity (heritability 0.79), and pods per plant (heritability 0.78). In addition, significant environmental effects were detected for several agronomic traits, including days to flowering, days to maturity, and yield-related traits such as pods per plant and 100-seed weight (**Table 2**), suggesting that both genetic constitution and growing environment play important roles. In fact, while determining the phenotypic and yield performances, the need to consider both factors in the selection of common bean genotypes is necessary, as was also reported by Pereira et al. (2017). Furthermore, a highly significant G×E interaction was observed for all the studied agronomic and phenological traits, indicating genotype responses across environments. This pattern is consistent with previous findings in common bean (Tigist et al., 2023; Gayosso Barragán et al., 2025) and underscores the importance of multi-environment testing in breeding programs. The high heritability estimates for yield-related phenological traits, such as days to flowering and physiological maturity, suggest that these traits are largely genetically determined and can be effectively transferred through breeding. Similar observations have been reported in earlier studies on common bean (Langat et al., 2019; Amongi et al., 2023a).

It is noteworthy that environmental factors influenced the performance of genotypes (**Figure 2**) as they were grown at locations that differed in precipitation and temperature. The location effect associated with climate variation evident in this study was similar to findings from the study by Pereira et al. (2017). The results from this study indicate that the less stressful growing environment in Namulonge, characterized by higher precipitation compared to Mubuku, resulted in shorter days to flowering (DF) and physiological maturity (DPM), and higher yield components, including seeds per pod (Seed_P), plot yield (PYLD), and 100-seed weight (Wt_100S). These findings signify precipitation as a crucial factor in common bean production. Lower yields in common beans due to frequent droughts were observed in similar studies conducted in northeast Brazil, the highlands of Mexico, and the eastern and southern African highlands from Ethiopia to South Africa (Beebe et al., 2013). These yield reductions are partly related to the physiological background of drought tolerance and to genotype responses to drought stress, which are important to consider when selecting genotypes for breeding programs. Accordingly, the use of genotypes carrying drought-tolerance genes adapted to drier climates, together with methods that enable accurate phenotyping of desired traits, is of critical importance. Nonetheless, further testing of these genotypes for adaptability and stability through multi-year and multi-environment field trials in Uganda is critically important. Likewise, significant differences in genotypic variances (**Table 2**) and the variable frequency distribution of BLUPs (**Figure 2**) for agronomic, phenological, and cooking quality traits highlight the broad genetic diversity of the common bean genotype panel in this study. Nonetheless, the significant effects of genotype and environment on cooking time, and of environment on water absorption, observed in this study, suggest that cooking quality traits are dependent on the growing environment (Katuuramu et al., 2020; Mvile et al., 2023; Jeffery et al., 2025). Quality related traits, such as cooking time, water uptake during cooking, etc., are important traits to consider, as common beans in Uganda and globally are used for human consumption, where processing (e.g. cooking) is extremely important to release nutrients and make them palatable (Uebersax et al., 2023; Asiimwe et al., 2024; Jeffery et al., 2025).

The low heritability for cooking time observed in this study indicates that a substantial proportion of the phenotypic variation of this trait is influenced by environmental factors, despite significant genotypic and environmental variance (P < 0.05). The non-significant genotype × environment interaction suggests that the relative performance of genotypes for this trait is generally consistent across environments. However, evaluation across multiple diverse growing environments is recommended to more accurately estimate the genetic contribution to cooking time, identify genotypic differences, and select genotypes with shorter cooking times, including stable performance across different climates. While the low G×E may allow assessment in fewer environments, as suggested by Katuuramu et al. (2020), adequate environmental representation remains important to improve the reliability of heritability estimates and to account for environmental influences on this complex trait. In the present study, seeds from the Mubuku field trials exhibited shorter cooking times (CT) than those from the Namulonge trials. This difference may reflect variation in the expression of genes governing cooking time across the two locations, which warrants further investigation. Cooking time is known to increase as the concentration of soluble compounds in the cotyledons decreases; hence, alleles associated with increased soluble compound content are likely to contribute to longer cooking times (Jeffery et al., 2025). This effect may be related to calcium-binding storage proteins and other calcium-containing compounds, such as phytates, and to higher levels of insoluble tannins and lignin in the cotyledon walls, which collectively influence the structural properties of the cotyledons (Jeffery et al., 2025) and, consequently, overall cooking quality.

Germplasm genetic diversity is essential to common bean improvement for different traits (Al-Khayri et al., 2019). The results of this study indicate high genetic diversity within the genotype collection with respect to the traits evaluated, except for water absorption capacity. Regarding water absorption capacity, the non-significant impact of the genotype effect observed in this study contrasts with the significant differences and high variability attributed to genotype in other studies (Diaz et al., 2021; Sadohara et al., 2022). One possible explanation could be related to the optimum soaking time. For example, common beans in this study were soaked for 18 hours, while other studies used soaking times of around 12 hours (Diaz et al., 2021; Sadohara et al., 2022). It is also known that the water absorption in common beans peaks around 11–14 hours of soaking (Corrêea et al., 2010), suggesting that the soaking duration used in this study may have been too long. As a result, water absorption capacity may have been measured after the genotypes had exceeded their peak absorption, potentially obscuring genotypic differences. For example, no significant differences in the performance of the genotypes were observed in wild *Vigna* legumes soaked for an extended duration of 24 hours (Harouna et al., 2019). Nonetheless, Amongi et al. (2023b) found significant genotype differences when common beans were soaked for 18 hours, and the study included a larger number (427) of genotypes than we had in this study. Similarly, other studies included large collections of genotypes, 295 (Sadohara et al., 2022) and over 400 genotypes Diaz et al. (2021), indicating a need for a larger sample collection. Therefore, in the future, studies with large collections of material as well as optimized soaking protocols are expected to better explore genetic-environmental interactions affecting hydration kinetics.

Days to flowering is an important trait to consider when breeding common beans for climate change resilience. Moreover, in this study, it was positively correlated with yield traits. Days to flowering, days to maturity, and pods per plant are traits related to seed yield, and such relationships have been reported in several crops, including those grown under drought stress conditions, such as legumes (Langat et al., 2019) and cereals (Lama et al., 2023). Furthermore, the traits such as pods per plant were suggested as selection criteria for high seed yield in common bean breeding for drought-prone environments, due to their high heritability (Langat et al., 2019). Drought exposes common bean plants to water stress during the most sensitive plant development stages, which in turn reduces pod filling, pods per plant, seeds per pod, and ultimately grain yield (Wasae, 2021; Karavidas et al., 2022). Early-maturing common beans were found to be more tolerant to drought stress; here, suggesting days to flowering as a key preferred trait of growers in the context of global climate change (Mukankusi et al., 2015; Nchanji et al., 2021). Hence, identifying genotypes that flower early, mature early, and maintain high yield potential under drought stress conditions is paramount.

The significant negative correlation found in this study between 100-seed weight and days to flowering, as well as the other yield-component traits, indicated that the small-seeded genotypes tend to take longer to flower. This finding is in line with previous studies, which found a negative correlation between 100-seed weight and yield components (Rana et al., 2015; Murube et al., 2019; Diaz et al., 2022). Seed yield-component traits, including 100-seed weight, number of pods per plant, and seeds per pod, are genetically controlled by multiple genes, primarily with additive effects (Blair et al., 2006; Hoyos-Villegas et al., 2016; Murube et al., 2019; Geravandi et al., 2020). Quantitative trait loci (QTL) alleles associated with a higher number of pods per plant were also found to be associated with smaller 100-seed weight or late flowering (Blair et al., 2006). According to Geravandi et al. (2020), the detected QTLs showed varying additive effects for different seed yield components: alleles at QTLs for seed weight and number of seeds per pod tended to decrease these traits, whereas alleles at QTLs for pods per plant and seeds per plant tended to increase seed weight and number of seeds per pod. Since these four traits are key contributors to overall seed yield, the results suggest that selecting alleles with favorable effects on the component traits that increase yield could enhance total seed production.

Strategies for common bean breeding in the changing climate era need to consider the national, regional, and international markets, as well as evolving agri-environmental conditions. Selecting promising genotypes that are stable not only in production outputs but also carry genes that control important pest and disease resistance, abiotic stress tolerance, and are end-user accepted is of utmost importance. From this study, we identified seven common bean genotypes: NAROBEAN6, SMR48, SCR60, SCR45, SCN20, KND100, and SCN744, that exhibited superior agronomic and phenological traits (e.g. days to flowering, days to physiological maturity, pods per plant, seeds per plant, seeds per pod, yield, 100-seed weight) as well as cooking quality (e.g. cooking time) across two contrasting growing environments. Several studies have identified superior performing common beans based on cooking time (Katuuramu et al., 2020; Sadohara et al., 2022; Mughi et al., 2017), on phenological and agronomic traits (Rana et al., 2015; Amongi et al., 2023a; Lama et al., 2023). The superior performing genotypes identified in this study can be used as sources of genes for end-user traits and/or be presented to breeders for further evaluation, e.g., in multiple traditional growing areas in different parts of Uganda. This step should also include farmers’ participation in the evaluation of candidate varieties, which greatly contribute to crop improvement, recognizing that farmers’ criteria of varietal selection extend beyond yield and stress tolerance/resistance to include consumption quality, marketability, suitability to the soil, and labor requirements for production (Weltzien and Christinck, 2017; Awio et al., 2018; Nanyonjo et al., 2024).

## 5 Conclusion

This study highlighted that traits such as seed color and size, days to flowering and physiological maturity, yield components (pods per plant, seeds per plant and per pod, 100-seed weight) and cooking time, are important for breeding common beans. These traits should be further evaluated for the promising genotypes in multi-year and multi-environment field trials.

Genotype and environment, as well as the genotype x environment interaction significantly influenced nearly all the studied traits, except water absorption capacity, which was influenced by only the growing environment. This study highlighted that red beans had a long cooking time, while white beans cooked faster, although profound analysis of seed coat thickness and composition of the seed should be conducted. Moreover, the phenological traits, *e.g.* days to flowering and physiological maturity are critical when common beans are grown in drought-stress environments. Seven common bean genotypes (NAROBEAN6, SMR48, SCR60, SCR45, SCN20, KND100, and SCN744) that exhibited superior performance in terms of desirable characteristics of the evaluated phenological, agronomic, and cooking quality traits should be further evaluated in climate resilience studies in multi- environments and in common bean improvement programs. It is noteworthy that a released variety, NAROBEAN6, was ranked among the superior genotypes out of 23 used in this study, which clearly shows a need for improvement of genetic material. Since the 100-seed weight was associated with genotype performance, this trait could be further tested during germplasm selection to breed, particularly for yield and cooking time. Follow-up studies across multiple locations, including the promising genotypes from this study are highly valuable to evaluate the genotypes’ responses to diverse climates, including those with severe droughts. In conclusion, the outcomes of this study support the breeding efforts to improve common beans, align farmer and consumer needs, and contribute to the sustainable development of food systems, energy efficiency, and improved household nutrition.

## Data Availability

The original contributions presented in the study are included in the article/**Supplementary Material**. Further inquiries can be directed to the corresponding author.

## Supplementary material

Supplementary Figure 1 Rainfall and temperature patterns in Mubuku and Namulonge across two seasons: season 1 (Sept 2023- December 2024), and season 2 (April-July 2024).

Supplementary Figure 2 Principal-component analysis (PCA) using BLUP values of traits of four categories, i.e., phenological traits: days to flowering (DF) and days to physiological maturity (DPM); yield components: seeds per pod (S_Pod), pods per plant (Pod_P), seeds per plant (Seed_P); plot yield (PYLD); cookability: cooking time (CT). The 199 genotypes were grouped according to the three clusters identified by the hierarchical clustering.
