# Supplementary figures and images for: From the field to the pot: phenological, agronomic, and cookability traits of common beans (Phaseolus vulgaris L.) grown in contrasting climatic regions in Uganda
Source: Front Plant Sci. 2026 Jun 17;17:1811268. doi: 10.3389/fpls.2026.1811268 (PMC13319034; doi:10.3389/fpls.2026.1811268)

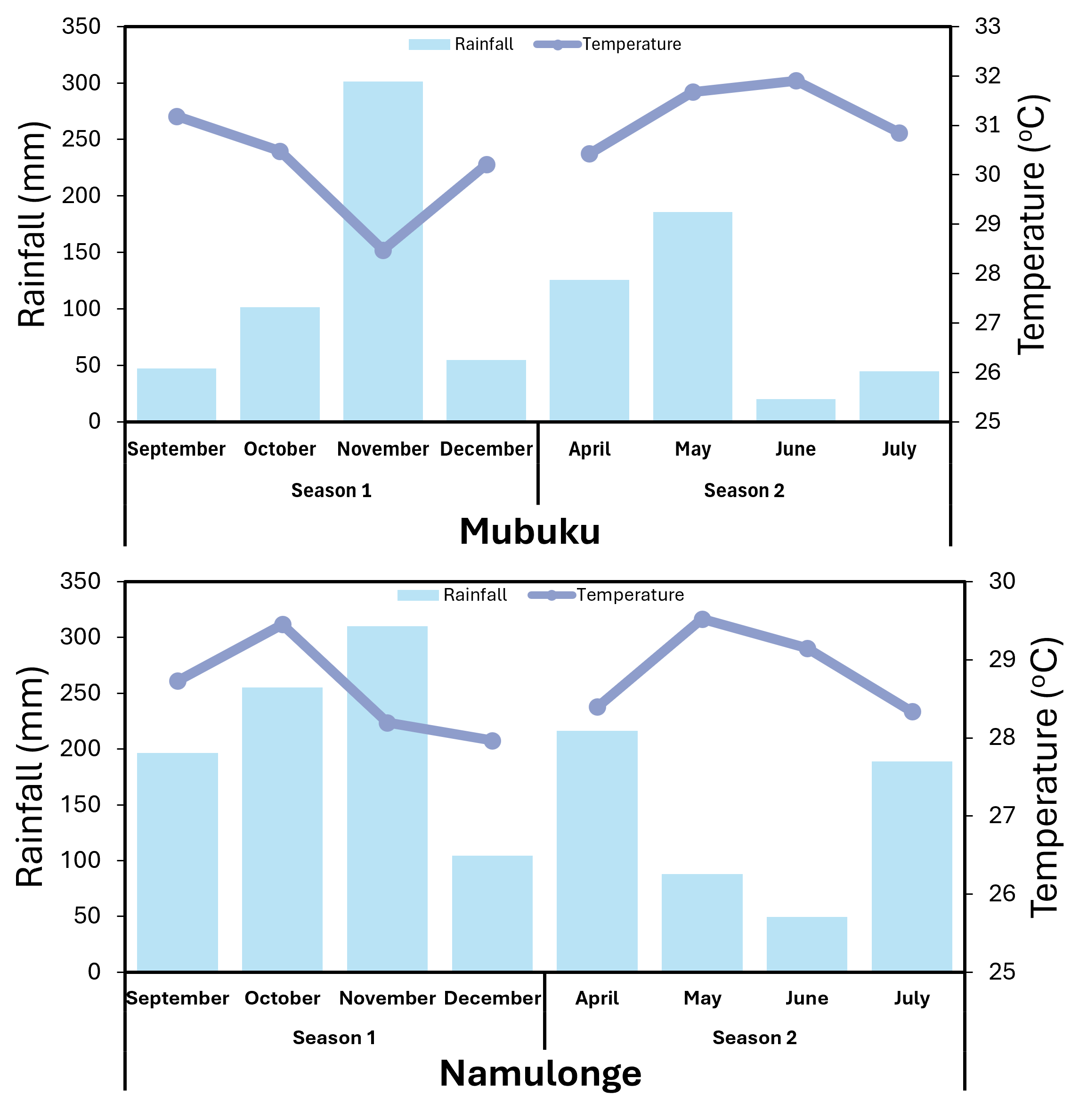

Supplement: Supplementary Figure 1 — Rainfall and temperature patterns in Mubuku and Namulonge across two seasons: season 1 (Sept 2023- December 2024), and season 2 (April-July 2024). [file Image1.tif]

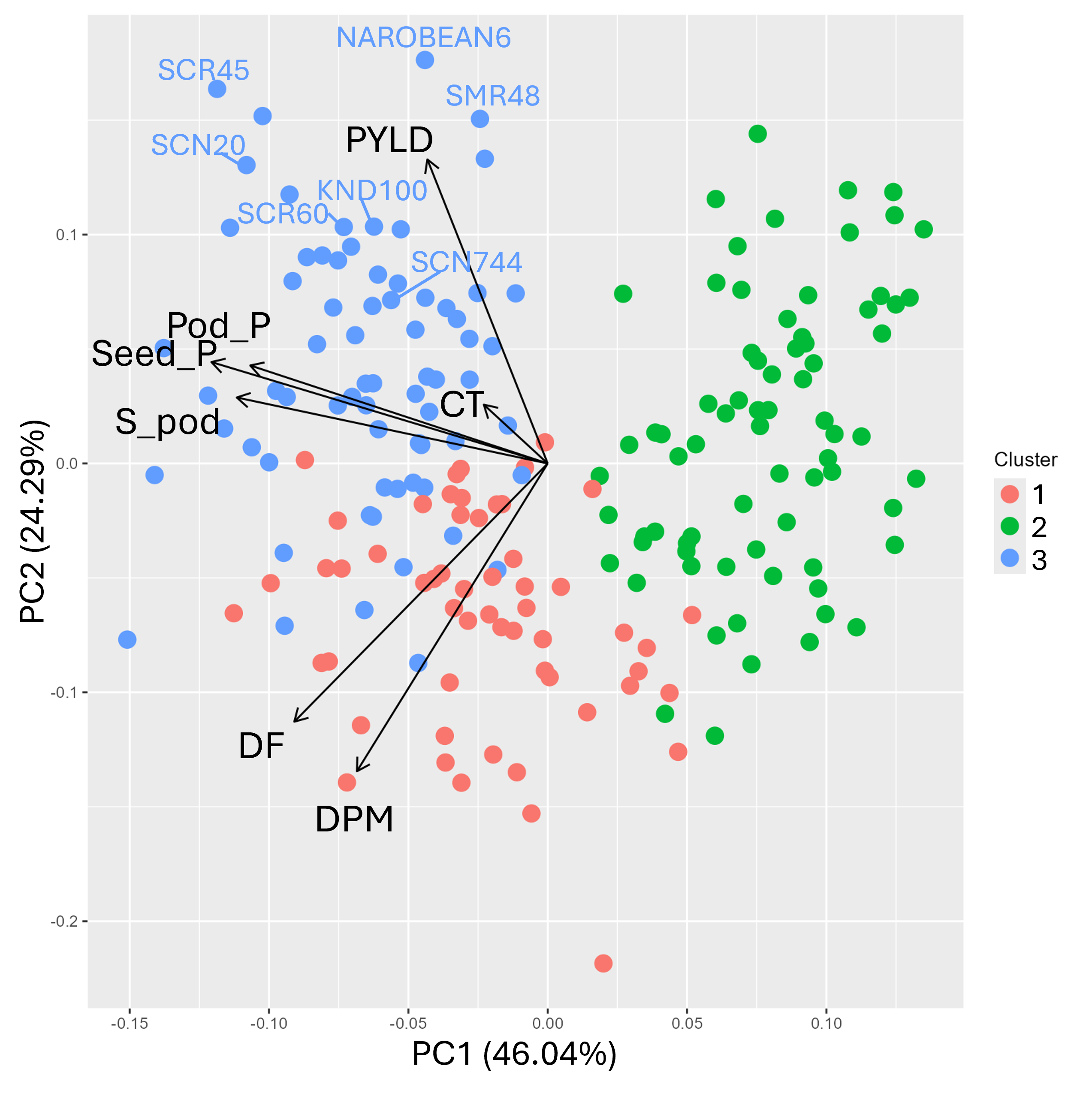

Supplement: Supplementary Figure 2 — Principal-component analysis (PCA) using BLUP values of traits of four categories, i.e., phenological traits: days to flowering (DF) and days to physiological maturity (DPM); yield components: seeds per pod (S_Pod), pods per plant (Pod_P), seeds per plant (Seed_P); plot yield (PYLD); cookability: cooking time (CT). The 199 genotypes were grouped according to the three clusters identified by the hierarchical clustering. [file Image2.tif]
