# Supplementary material for: From the field to the pot: phenological, agronomic, and cookability traits of common beans (Phaseolus vulgaris L.) grown in contrasting climatic regions in Uganda
Source: Front Plant Sci. 2026 Jun 17;17:1811268. doi: 10.3389/fpls.2026.1811268 (PMC13319034; doi:10.3389/fpls.2026.1811268)
Supplement: Supplementary file 3 [file Table1.docx]

Supplementary Material

**Supplementary Table 1**. Metadata of the common bean genotypes used in the study

| **Entry** | **Market class** | **Trait** | **Source** |
| --- | --- | --- | --- |
| SEC38 | White | Drought | CIAT |
| GRR21 | Red | Drought | CIAT |
| BFS88 | Red | Yield | CIAT |
| ACC15 | Sugar | CBB_moderate | CIAT |
| SMC137 | Cream | Fe/Zn | CIAT |
| SMC158 | Yellow | Fe/Zn | CIAT |
| SMR221 | Medium Red | Fe/Zn | CIAT |
| ICNBunsixSxB405/1C-1C-1C-B | white | PRR/ALS | CIAT |
| SMC261 | Medium Cream | Fe/Zn | CIAT |
| BFS113 | Red |  | CIAT |
| BFS98 | Red | Yield | CIAT |
| HTA28 | Large Pink |  | CIAT |
| SMC147 | Kablanketi | Fe/Zn | CIAT |
| DAA313 | Large Sugar |  | CIAT |
| SMC16 | White | Fe/Zn | CIAT |
| RWR2154 | Sugar | Early maturity, yield, Fe/Zn | CIAT |
| UGK85 | Red mottled | Fe/Zn | CIAT |
| AAB_007 | Black | ALS | CIAT |
| DAA21 | Large Red Mottled |  | CIAT |
| NUA608 | Red | Fe/Zn | CIAT |
| MAB_349 | Red | ALS | CIAT |
| BFS143 | Red | Yield | CIAT |
| DAB13 | Red | Drought | CIAT |
| ADP-441 | Cream | Resistant to PRR/FRR | CIAT |
| SMR197 | Small Red | Fe/Zn | CIAT |
| SEC43 | Kablanketi | Drought | CIAT |
| NUA712 | Red mottled | Fe/Zn | CIAT |
| MAZ34 | Red mottled | Virus | CIAT |
| ACC28 | Red mottled | CBB_moderate | CIAT |
| ADP-58 | Red | FRR/PRR | CIAT |
| BFS142 | Red | Yield | CIAT |
| BFS87 | Red | Yield | CIAT |
| RW1180 | Red | Fe/Zn | CIAT |
| MAC44 | Red Mottled | Yield, Fe/Zn | CIAT |
| BFS95 | Red | Yield | CIAT |
| SMR268 | Small Dark Red | Fe/Zn | CIAT |
| BFS86 | Red | Yield | CIAT |
| NUA607 | Red | Fe/Zn | CIAT |
| SMR130 | Red | Fe/Zn | CIAT |
| SEQ1003 | Red mottled | Drought | CIAT |
| HTA31 | Large Red Mottled |  | CIAT |
| NUA702 | Red mottled | Fe/Zn | CIAT |
| NUS16 | MediumCarioca | Fe/Zn | CIAT |
| DAA305 | Large Red Mottled |  | CIAT |
| ACC27 | Red | CBB_moderate | CIAT |
| KND41 | Brown | Cooking time | CIAT |
| SMN115 | Medium Black | Fe/Zn | CIAT |
| SCN11 | Black | Early maturity, yield, tolerant to anthracnose, BCMV, drought, Fe/ Zn | CIAT |
| ICNBunsixSxB405/7C-1C-1C-30 | white | PRR/ALS | CIAT |
| SMR53 | Red | Fe/Zn | CIAT |
| ICNBunsixSxB405/4C-1C-1C-88 | white | PRR/FRR/ ALS | CIAT |
| SMN105 | Medium Black | Fe/Zn | CIAT |
| DAA328 | Large Red Mottled |  | CIAT |
| GAB5 | Large Red |  | CIAT |
| ACC:182054/IDPUEBLA152 | Black | FRR/PRR | CIAT |
| BFS117 | Red | Generally low disease | CIAT |
| KND86 | Red | Cooking time | CIAT |
| SMR118 | DarkRed | Fe/Zn | CIAT |
| BFS100 | Red | Yield | CIAT |
| SMN111 | Medium Black | Fe/Zn | CIAT |
| KFRR37b | Red mottled | Virus, FRR | CIAT |
| GRR23 | Red | Drought | CIAT |
| KARP22 | Cream | PRR, FRR | CIAT |
| GRR29 | Pink | Drought | CIAT |
| ADP-438 | Red mottled | FRR/PRR | CIAT |
| SMC151 | Kablanketi | Fe/Zn | CIAT |
| RAZ44-Alubia | white | PRR/ALS | CIAT |
| SMG17 | Medium Pink | Fe/Zn | CIAT |
| NUS21 | Light Red | Fe/Zn | CIAT |
| DAB216 | Sugar | Drought | CIAT |
| GGB9 | Red | Drought | CIAT |
| 108958 | White | FRR/PRR | CIAT |
| DAA309 | Large Red Mottled |  | CIAT |
| GAN11 | Medium Red |  | CIAT |
| BFS119 | Black | Yield | CIAT |
| NABE3 | Small Red | Resistant to BCMV and anthracnose | NARO |
| DAN15 | Red | Fe/Zn | CIAT |
| DAB520 | Red | Drought | CIAT |
| MAZ42 | Red mottled | Virus | CIAT |
| SMR48 | Red | Fe/Zn | CIAT |
| SMR229 | Medium Red | Fe/Zn | CIAT |
| CMKFS75 | Cream | FRR, yield | CIAT |
| SMN122 | Small Black | Fe/Zn | CIAT |
| SMR128 | Red | Fe/Zn | CIAT |
| KFRR138 | Red mottled | Virus, FRR | CIAT |
| SMC163 | White | Fe/Zn | CIAT |
| KNG14-30 | Red | Virus, CBB | CIAT |
| UGK39 | Pink | Fe/Zn | CIAT |
| GAB1 | Large Sugar |  | CIAT |
| DAB917 | Red mottled | Drought | CIAT |
| SAB618 | Large Red Mottled |  | CIAT |
| RWR2245 | Red Mottled | Early maturity, yield, Fe/Zn | CIAT |
| K11714 | Pink | FRR/PRR | CIAT |
| ADP-517 | Cream | ALS/FRR/PRR | CIAT |
| DAA14 | Large Red |  | CIAT |
| SMC201 | Purple | Cooking time | CIAT |
| DAB30 | Sugar | Drought | CIAT |
| JESCA | Purple |  | CIAT |
| AAB_005_b | Red | ALS | CIAT |
| CAL96 | Red Mottled | Early maturity, yield, tolerant to anthracnose, BCMV | CIAT |
| DAA326 | Medium Red Mottled |  | CIAT |
| NABE16 | Medium Red Mottled | Early maturity, yield, tolerant to anthracnose, swelling capacity | NARO |
| NUA671 | Sugar | Fe/Zn | CIAT |
| NABE11 | Large Sugar | Cooking time, resistant to CBB | NARO |
| NABE20 | Large Sugar | Early maturity, yield, tolerant to anthracnose, BCMV | NARO |
| KG71-42 | Red | Drought | CIAT |
| KCN9 | White | Cooking time | CIAT |
| BFS84 | Red | Yield | CIAT |
| NUA668 | Red mottled | Fe/Zn | CIAT |
| BFS92 | Red | Yield | CIAT |
| ACC:110149/ID:VERANO | White | CBB_moderate | CIAT |
| DAB599 | Red | Drought | CIAT |
| F14Population(23) | White | ALS | CIAT |
| NAROBEAN3 | Medium Yellow | Early maturity, yield, Fe and Zn | NARO |
| KFRR275 | Red mottled | Virus, FRR | CIAT |
| KND85 | Red | Cooking time | CIAT |
| KCN12 | Sugar | Cooking time | CIAT |
| SMC160 | Yellow | Fe/Zn | CIAT |
| NAROBEAN2 | Medium Red Mottled | Early maturity, yield, Fe and Zn | NARO |
| SMC258 | Medium Brown | Fe/Zn | CIAT |
| SER125 | Medium Red |  | CIAT |
| SMC255 | Medium Yellow | Fe/Zn | CIAT |
| NABE15 | Medium Sugar | Yield, tolerant to anthracnose, swelling capacity | NARO |
| NAROBEAN7 | Medium Black | Early maturity, yield, tolerant to anthracnose, BCMV, drought, Fe/Zn | NARO |
| N11277 | White | FRR/PRR | CIAT |
| SER128 | Red | Drought | CIAT |
| SMR245 | Medium Red | Fe/Zn | CIAT |
| NABE23 | Large Sugar | Early maturity, yield, tolerant to anthracnose, BCMV, swelling capacity | NARO |
| AAB_001_b | Red | ALS | CIAT |
| ETHOPIA1 | Red | Early maturity, resistance to angular leaf spot, CBB | CIAT |
| NUA35 | Red mottled | Fe/Zn | CIAT |
| BFS137 | Red | Yield | CIAT |
| SMC22 | White | Fe/Zn | CIAT |
| SMC196 | Purple | Cooking time | CIAT |
| DAB290 | Red mottled | Drought | CIAT |
| CMKFS71 | Purple mottled | FRR, yield | CIAT |
| MAB_373 | Red | ALS | CIAT |
| SCR45 | Red | FRR/PRR | CIAT |
| CMKFS132 | White | FRR, yield | CIAT |
| SCR60 | Red | Drought | CIAT |
| KATB1 | Medium Yellow |  | CIAT |
| BFS99 | Red | Yield | CIAT |
| CMKFS105 | Cream | FRR, yield | CIAT |
| NABE12C | Sugar | Cooking time, resistant to root rot, yield, taste | NARO |
| DAB919 | Red mottled | Drought | CIAT |
| NUA682 | Red mottled | Fe/Zn | CIAT |
| ROBA1 | Small Cream | Nutrition line | CIAT |
| SMR122 | Red | Cooking time | CIAT |
| CMKFS88 | Black | FRR, yield | CIAT |
| SMC215 | Pink mottled | FRR/PRR | CIAT |
| BAT477N | Cream | Yield | CIAT |
| NUA605 | Red | Fe/Zn | CIAT |
| K131 | Small Carioca | Yield, resistance to CBMV, and anthracnose | CIAT |
| K132 | Large Red Mottled | Early maturity, yield, tolerant to anthracnose, BCMV | CIAT |
| SMR44 | Red | Fe/Zn | CIAT |
| SCN20 | Black | Drought | CIAT |
| SAB712 | Medium White |  | CIAT |
| F10blockselnewbelfa-63 | Yellow | Drought | CIAT |
| ETSNAP16 | white | PRR/ALS | CIAT |
| SMC28 | Yellow | Fe/Zn | CIAT |
| NABE22 | Medium Purple Mottled | Early maturity, yield, tolerant to anthracnose, BCMV, swelling capacity | NARO |
| KCN10 | White | Cooking time | CIAT |
| RAZ-11 | white | PRR/FRR/ ALS | CIAT |
| DAB249 | Red mottled | Drought | CIAT |
| NUA704 | Red mottled | Fe/Zn | CIAT |
| KFRR39 | Red mottled | Virus, FRR | CIAT |
| SEQ1027 | Sugar | Drought | CIAT |
| DAA336 | Large Red Mottled |  | CIAT |
| DAN9 | RedKidney | Fe/Zn | CIAT |
| KG111-26 | Red | Drought | CIAT |
| NABE17 | Large Red Mottled | Early maturity, yield, tolerant to anthracnose, BCMV | NARO |
| DAA332 | Large Red Mottled |  | CIAT |
| MIB465 | Black | Regional high iron check | CIAT |
| DAB34 | Sugar | Drought | CIAT |
| NABE14 | Medium Red | Yield, tolerant to root rot disease | NARO |
| NABE19 | Large Red Mottled | Early maturity, yield, tolerant to anthracnose, BCMV | NARO |
| NABE21 | Small Sugar | Early maturity, yield, tolerant to anthracnose, BCMV | NARO |
| SCN744 | Black | Drought | CIAT |
| ALB169 | Red | FRR/PRR/Yield | CIAT |
| CMKFS109 | Black | FRR, yield | CIAT |
| DAB91 | Red | Drought | CIAT |
| G10677 | Brown | Drought | CIAT |
| KFRR287 | Red mottled | FRR | CIAT |
| DAA323 | Large Red |  | CIAT |
| ACC14 | Red | CBB_resistant | CIAT |
| DAB291 | Red mottled | Drought | CIAT |
| DAA303 | Large Sugar |  | CIAT |
| DAA339 | Large Red Mottled |  | CIAT |
| KG6-10 | Red | Drought | CIAT |
| SMC146 | Medium Kablanketi | Fe/Zn | CIAT |
| KND100 | Dark Red | Cooking time | CIAT |
| MAB_354 | Red | ALS | CIAT |
| NUS5 | Red | Fe/Zn | CIAT |
| VAX1 | Red | ALS | CIAT |
| NE34-12-42 | Black | FRR/PRR | CIAT |
| KNG14-43 | Red | Virus, CBB | CIAT |
| KCN62 | White | Cooking time | CIAT |
| ETSNAP12 | white | PRR/ALS | CIAT |
| CMKFS114 | White | FRR, yield | CIAT |
| SSIN1174 | Pink | Drought | CIAT |
| ACC31 | Red | CBB_moderate | CIAT |
| DAA6 | Red | Drought | CIAT |
| SARBYT2 | Red | Drought | CIAT |
| SMR127 | Red | Fe/Zn | CIAT |
| SMR106 | Red | Fe/Zn | CIAT |
| DAB192 | Sugar | Drought | CIAT |
| SSIN943 | Red mottled | Drought | CIAT |
| SMC159 | Red | Cooking time | CIAT |
| GGB2 | Red | Drought | CIAT |
| CMKFS122 | White | FRR, yield | CIAT |
| KND90 | Dark Red | Cooking time | CIAT |
| SMR117 | Red | Cooking time | CIAT |
| SMC207 | Red Mottled | Cooking time | CIAT |
| SMC168 | Cream | Cooking time | CIAT |
| SMR133 | Red | Cooking time | CIAT |
| SMC171 | Brown | Cooking time | CIAT |
| SEC93 | Medium Pink | Fe/Zn | CIAT |
| KND25 | Red | Cooking time | CIAT |
| NUS31 | Light Red | Fe/Zn | CIAT |
| SMC142 | Large Purple Mottled | Fe/Zn | CIAT |
| KCN64 | White | Cooking time | CIAT |
| NUA517 | Red mottled | Fe/Zn | CIAT |
| ETSNAP31 | white | PRR/ALS | CIAT |
| RW547 | Brown | Fe/Zn | CIAT |
| KND36 | Brown | Cooking time | CIAT |
| DAA327 | Medium Red Mottled |  | CIAT |
| ALB8 | Red | FRR/PRR/Yield | CIAT |
| DAA301 | Large Sugar |  | CIAT |
| BFS104 | Red | Yield | CIAT |
| NAROBEAN6 | Small red | Early maturity, yield, tolerant to anthracnose, BCMV, drought, Fe and Zn | NARO |
| SMC24 | White | ALS/FRR/PRR | CIAT |
| KNG17-24 | Purple | Virus, CBB | CIAT |
| NUA711 | Red mottled | Fe/Zn | CIAT |
| NABE4 | Large Red Mottled | Yield, resistance to CBMV, anthracnose, drought | NARO |
| NAROBEAN1 | Large Sugar | Early maturity, yield, Fe/Zn | NARO |
| SCR26 | Small red | Early maturity, yield, tolerant to anthracnose, BCMV, drought, Fe/Zn | CIAT |
| NUA595 | Red | Fe/Zn | CIAT |
| SMC141 | Medium Purple Mottled | Fe/Zn | CIAT |
| MEX54 | Pink | ALS | CIAT |
| AWASH1 | Small White |  | CIAT |
| DAA333 | Large Sugar |  | CIAT |
| NABE13 | Large Red Kidney | Yield, tolerant to root rot disease | NARO |
| NUS27 | Medium Sugar | Fe/Zn | CIAT |
| CMKFS24 | Cream | FRR, yield | CIAT |
| DAA129 | Large Red |  | CIAT |
| HTA29 | Large Red Mottled |  | CIAT |
| SEC90 | Medium Brown | Fe/Zn | CIAT |

CBB = common bacterial blight; Fe = iron; Zn = zinc; ALS = angular leaf spot; BCMV = bean common mosaic virus; PRR = pythium root rot; FRR = fusarium root rot.

**Supplementary Table 2.** Summary statistics showing the performance of released varieties and breeding lines for the evaluated traits

| Summary statistics | DF | DPM | Pod_P | Seed_P | S_pod | PYLD | CT | Wt_100S |
| --- | --- | --- | --- | --- | --- | --- | --- | --- |
| Released varieties |  |  |  |  |  |  |  |  |
| Mean | 37.29 | 67.56 | 7.97 | 25.98 | 3.61 | 1283.56 | 71.51 | 30.75 |
| Min | 34.83 | 64.35 | 5.67 | 17.28 | 2.97 | 939.44 | 66.09 | 22.3 |
| Max | 41.33 | 71.97 | 11.72 | 42.86 | 4.26 | 1808.85 | 87.16 | 36.33 |
| Breeding  lines |  |  |  |  |  |  |  |  |
| Mean | 38.39 | 69.14 | 9.01 | 33.00 | 3.94 | 1240.85 | 72.55 | 27.08 |
| Min | 32.41 | 62.66 | 5.45 | 16.96 | 2.97 | 876.29 | 63.25 | 17.07 |
| Max | 43.34 | 75.2 | 15.74 | 60.11 | 5.17 | 1879.46 | 86.62 | 43.05 |

**Supplementary Table 3.** Best Linear Unbiased Predictors (BLUPs) based on the ranking of four trait categories i.e., phenological, yield components, plot yield, and cookability traits for 199 common bean genotypes grown at two locations across two seasons, along with their corresponding 100-seed size and clusters, which were determined according to variation of the evaluated traits.

| Genotype | Phenological | Yield components | Grain yield | Cookability | Overall ranking | Wt_100S | Cluster | Type |
| --- | --- | --- | --- | --- | --- | --- | --- | --- |
| NAROBEAN6 | 28.50 | 32.33 | 3.00 | 36.00 | 24.96 | 24.48 | 3 | Released variety |
| SMR48 | 24.50 | 47.00 | 11.00 | 37.00 | 29.88 | 25.86 | 3 | Breeding line |
| SCR60 | 75.00 | 38.00 | 7.00 | 20.00 | 35.00 | 25.04 | 3 | Breeding line |
| SCR45 | 42.50 | 12.00 | 9.00 | 96.00 | 39.88 | 23.45 | 3 | Breeding line |
| SCN20 | 78.00 | 13.00 | 4.00 | 75.00 | 42.5 | 26.24 | 3 | Breeding line |
| KND100 | 72.00 | 36.00 | 14.00 | 52.00 | 43.5 | 25.65 | 3 | Breeding line |
| SCN744 | 104.50 | 51.33 | 5.00 | 33.00 | 48.46 | 26.03 | 3 | Breeding line |
| GRR21 | 92.00 | 38.33 | 2.00 | 68.00 | 50.08 | 29.94 | 3 | Breeding line |
| NAROBEAN1 | 53.50 | 117.33 | 17.00 | 14.00 | 50.46 | 34.08 | 2 | Released variety |
| GRR23 | 74.00 | 33.33 | 24.00 | 74.00 | 51.33 | 29.56 | 3 | Breeding line |
| DAA301 | 21.50 | 124.33 | 19.00 | 48.00 | 53.21 | 40.34 | 2 | Breeding line |
| SCN11 | 106.00 | 16.00 | 12.00 | 84.00 | 54.5 | 23.91 | 3 | Breeding line |
| SER128 | 55.00 | 20.00 | 43.00 | 102.00 | 55.00 | 26.56 | 3 | Breeding line |
| SMN111 | 64.00 | 64.00 | 52.00 | 44.00 | 56.00 | 23.96 | 3 | Breeding line |
| KND25 | 129.00 | 41.33 | 1.00 | 53.00 | 56.08 | 26.21 | 3 | Breeding line |
| SMN122 | 108.50 | 32.67 | 16.00 | 69.00 | 56.54 | 25.22 | 3 | Breeding line |
| DAA339 | 7.00 | 128.67 | 23.00 | 73.00 | 57.92 | 40.14 | 2 | Breeding line |
| DAA313 | 4.5 | 144.67 | 40.00 | 45.00 | 58.54 | 37.22 | 2 | Breeding line |
| BFS98 | 65.5 | 41.67 | 22.00 | 106.00 | 58.79 | 24.46 | 3 | Breeding line |
| KND41 | 184.00 | 25.67 | 6.00 | 24.00 | 59.92 | 24.43 | 3 | Breeding line |
| SSIN943 | 39.00 | 45.67 | 75.00 | 80.00 | 59.92 | 22.34 | 3 | Breeding line |
| SMC159 | 48.00 | 14.33 | 18.00 | 166.00 | 61.58 | 25.08 | 3 | Breeding line |
| SEC43 | 82.00 | 48.67 | 35.00 | 90.00 | 63.92 | 25.47 | 3 | Breeding line |
| NABE23 | 42.00 | 144.33 | 65.00 | 6.00 | 64.33 | 29.72 | 2 | Released variety |
| K132 | 68.50 | 138.00 | 47.00 | 5.00 | 64.63 | 36.06 | 2 | Breeding line |
| SMC137 | 113.50 | 64.67 | 79.00 | 12.00 | 67.29 | 22.20 | 1 | Breeding line |
| DAA336 | 32.00 | 120.33 | 70.00 | 49.00 | 67.83 | 39.57 | 2 | Breeding line |
| KNG14-30 | 83.5 | 44.00 | 82.00 | 62.00 | 67.88 | 27.04 | 3 | Breeding line |
| DAA328 | 8.00 | 154.00 | 56.00 | 55.00 | 68.25 | 37.91 | 2 | Breeding line |
| DAB599 | 83.00 | 134.00 | 48.00 | 9.00 | 68.5 | 40.92 | 2 | Breeding line |
| SMR229 | 99.00 | 62.67 | 31.00 | 86.00 | 69.67 | 27.61 | 3 | Breeding line |
| SMC255 | 92.5 | 35.67 | 10.00 | 141.00 | 69.79 | 22.67 | 3 | Breeding line |
| RWR2154 | 69.00 | 111.00 | 87.00 | 13.00 | 70.00 | 33.74 | 2 | Breeding line |
| NABE13 | 103.5 | 119.00 | 46.00 | 16.00 | 71.13 | 35.46 | 2 | Released variety |
| DAN9 | 46.50 | 155.33 | 67.00 | 17.00 | 71.46 | 33.19 | 2 | Breeding line |
| CMKFS88 | 105.00 | 67.67 | 54.00 | 64.00 | 72.67 | 24.55 | 3 | Breeding line |
| ALB8 | 97.50 | 33.67 | 92.00 | 71.00 | 73.54 | 24.66 | 3 | Breeding line |
| KND90 | 122.00 | 31.33 | 13.00 | 129.00 | 73.83 | 25.25 | 3 | Breeding line |
| BFS84 | 78.50 | 26.33 | 98.00 | 95.00 | 74.46 | 26.31 | 3 | Breeding line |
| DAB290 | 19.00 | 149.00 | 91.00 | 39.00 | 74.5 | 29.14 | 2 | Breeding line |
| SMR53 | 65.50 | 34.67 | 41.00 | 157.00 | 74.54 | 23.56 | 3 | Breeding line |
| GGB9 | 76.00 | 40.67 | 27.00 | 162.00 | 76.42 | 29.31 | 3 | Breeding line |
| SMG17 | 12.00 | 61.33 | 49.00 | 78.00 | 77.08 | 25.33 | 3 | Breeding line |
| SMC258 | 118.00 | 83.33 | 94.00 | 19.00 | 78.58 | 25.04 | 1 | Breeding line |
| ROBA1 | 136.00 | 18.00 | 97.00 | 67.00 | 79.5 | 18.87 | 1 | Breeding line |
| SMR44 | 68.00 | 57.00 | 39.00 | 155.00 | 79.75 | 24.94 | 3 | Breeding line |
| DAA323 | 47.00 | 186.00 | 29.00 | 58.00 | 80.00 | 43.00 | 2 | Breeding line |
| SMC261 | 40.5 | 39.67 | 114.00 | 132.00 | 81.54 | 24.52 | 3 | Breeding line |
| BFS104 | 130.00 | 16.00 | 55.00 | 128.00 | 82.25 | 27.13 | 3 | Breeding line |
| SMR268 | 113.50 | 69.33 | 25.00 | 122.00 | 82.46 | 28.34 | 3 | Breeding line |
| NABE11 | 38.50 | 175.67 | 84.00 | 34.00 | 83.04 | 36.11 | 2 | Released variety |
| DAA309 | 26.50 | 128.33 | 45.00 | 135.00 | 83.71 | 36.34 | 2 | Breeding line |
| SCR26 | 122.50 | 49.67 | 58.00 | 105.00 | 83.79 | 24.91 | 3 | Breeding line |
| NABE16 | 47.00 | 83.33 | 8.00 | 199.00 | 84.33 | 36.33 | 3 | Released variety |
| KND36 | 187.00 | 33.67 | 69.00 | 50.00 | 84.92 | 25.34 | 1 | Breeding line |
| DAB917 | 38.00 | 155.33 | 71.00 | 76.00 | 85.08 | 36.82 | 2 | Breeding line |
| HTA31 | 28.50 | 161.00 | 86.00 | 65.00 | 85.13 | 36.33 | 2 | Breeding line |
| ACC27 | 112.50 | 158.00 | 59.00 | 15.00 | 86.13 | 35.83 | 2 | Breeding line |
| SMC28 | 186.00 | 74.33 | 34.00 | 51.00 | 86.33 | 25.93 | 1 | Breeding line |
| AAB_005_b | 63.00 | 126.00 | 77.00 | 83.00 | 87.25 | 27.71 | 2 | Breeding line |
| DAA333 | 11.50 | 136.00 | 123.00 | 82.00 | 88.13 | 35.12 | 2 | Breeding line |
| DAA303 | 3.00 | 161.00 | 60.00 | 130.00 | 88.5 | 37.07 | 2 | Breeding line |
| DAA326 | 16.00 | 150.33 | 28.00 | 160.00 | 88.58 | 37.47 | 2 | Breeding line |
| SEQ1003 | 91.50 | 143.00 | 112.00 | 8.00 | 88.63 | 28.34 | 2 | Breeding line |
| SSIN1174 | 54.00 | 42.67 | 76.00 | 183.00 | 88.92 | 21.58 | 3 | Breeding line |
| BFS143 | 101.50 | 76.33 | 118.00 | 60.00 | 88.96 | 24.66 | 1 | Breeding line |
| NUA517 | 62.50 | 153.33 | 37.00 | 103.00 | 88.96 | 31.02 | 2 | Breeding line |
| ALB169 | 101.00 | 58.00 | 72.00 | 125.00 | 89.00 | 22.78 | 3 | Breeding line |
| BFS100 | 101.50 | 37.67 | 73.00 | 144.00 | 89.04 | 26.44 | 3 | Breeding line |
| NABE3 | 181.50 | 55.33 | 61.00 | 59.00 | 89.21 | 22.30 | 1 | Released variety |
| SEC90 | 141.50 | 85.00 | 124.00 | 7.00 | 89.38 | 26.42 | 1 | Breeding line |
| DAN15 | 39.50 | 176.67 | 105.00 | 40.00 | 90.29 | 37.35 | 2 | Breeding line |
| HTA29 | 32.50 | 170.67 | 134.00 | 25.00 | 90.54 | 34.29 | 2 | Breeding line |
| SMR106 | 119.00 | 75.33 | 81.00 | 88.00 | 90.83 | 22.37 | 1 | Breeding line |
| NAROBEAN7 | 103.50 | 50.67 | 26.00 | 185.00 | 91.29 | 23.34 | 3 | Released variety |
| SMC146 | 84.00 | 79.33 | 32.00 | 171.00 | 91.58 | 25.43 | 3 | Breeding line |
| SMC147 | 112.50 | 105.00 | 51.00 | 100.00 | 92.13 | 24.82 | 3 | Breeding line |
| KG111-26 | 106.50 | 27.00 | 50.00 | 187.00 | 92.63 | 20.91 | 3 | Breeding line |
| SEC38 | 150.50 | 19.33 | 78.00 | 127.00 | 93.71 | 23.53 | 3 | Breeding line |
| SARBYT2 | 43.50 | 65.67 | 93.00 | 174.00 | 94.04 | 21.29 | 3 | Breeding line |
| 108958 | 152.50 | 69.00 | 129.00 | 26.00 | 94.13 | 20.86 | 1 | Breeding line |
| GAB1 | 91.00 | 131.67 | 107.00 | 47.00 | 94.17 | 29.38 | 2 | Breeding line |
| GAN11 | 48.50 | 171.00 | 116.00 | 42.00 | 94.38 | 29.18 | 2 | Breeding line |
| SMC160 | 162.50 | 68.00 | 36.00 | 111.00 | 94.38 | 25.03 | 3 | Breeding line |
| NABE19 | 67.00 | 129.00 | 66.00 | 116.00 | 94.5 | 32.58 | 2 | Released variety |
| SER125 | 72.00 | 87.33 | 126.00 | 93.00 | 94.58 | 23.61 | 1 | Breeding line |
| KNG14-43 | 96.50 | 45.33 | 113.00 | 124.00 | 94.71 | 24.01 | 3 | Breeding line |
| DAA327 | 8.00 | 187.67 | 33.00 | 151.00 | 94.92 | 39.03 | 2 | Breeding line |
| SMC158 | 146.50 | 69.00 | 62.00 | 104.00 | 95.38 | 23.94 | 1 | Breeding line |
| NAROBEAN2 | 60.50 | 154.33 | 140.00 | 27.00 | 95.46 | 30.63 | 2 | Released variety |
| GAB5 | 20.00 | 151.33 | 80.00 | 136.00 | 96.83 | 34.46 | 2 | Breeding line |
| SEC93 | 127.50 | 105.00 | 42.00 | 113.00 | 96.88 | 23.21 | 1 | Breeding line |
| K131 | 162.50 | 25.67 | 20.00 | 181.00 | 97.29 | 20.18 | 3 | Breeding line |
| KND85 | 137.50 | 43.67 | 38.00 | 172.00 | 97.79 | 24.39 | 3 | Breeding line |
| SMC151 | 152.00 | 66.00 | 15.00 | 159.00 | 98.00 | 26.28 | 3 | Breeding line |
| NABE15 | 30.00 | 143.67 | 83.00 | 137.00 | 98.42 | 32.21 | 2 | Released variety |
| SMR118 | 172.00 | 39.33 | 30.00 | 153.00 | 98.58 | 24.83 | 3 | Breeding line |
| RW547 | 88.00 | 111.00 | 99.00 | 99.00 | 99.25 | 24.03 | 1 | Breeding line |
| KG71-42 | 83.50 | 30.67 | 90.00 | 194.00 | 99.54 | 23.92 | 3 | Breeding line |
| DAA14 | 7.50 | 164.67 | 115.00 | 115.00 | 100.54 | 35.22 | 2 | Breeding line |
| GRR29 | 159.00 | 48.00 | 21.00 | 176.00 | 101.00 | 27.83 | 3 | Breeding line |
| DAB192 | 76.00 | 150.33 | 160.00 | 18.00 | 101.08 | 28.77 | 2 | Breeding line |
| NUA605 | 132.00 | 169.00 | 102.00 | 2.00 | 101.25 | 29.42 | 2 | Breeding line |
| SAB712 | 51.50 | 150.67 | 184.00 | 21.00 | 101.79 | 24.75 | 2 | Breeding line |
| ACC:182054/IDPUEBLA152 | 121.50 | 8.00 | 122.00 | 156.00 | 101.88 | 19.92 | 3 | Breeding line |
| DAA305 | 2.00 | 171.67 | 104.00 | 133.00 | 102.67 | 34.84 | 2 | Breeding line |
| DAA21 | 9.00 | 181.67 | 95.00 | 126.00 | 102.92 | 43.05 | 2 | Breeding line |
| MAZ34 | 25.00 | 141.33 | 110.00 | 140.00 | 104.08 | 30.50 | 2 | Breeding line |
| DAB520 | 23.50 | 151.00 | 106.00 | 138.00 | 104.63 | 33.17 | 2 | Breeding line |
| HTA28 | 18.00 | 141.67 | 63.00 | 196.00 | 104.67 | 33.23 | 2 | Breeding line |
| RWR2245 | 85.50 | 134.33 | 136.00 | 63.00 | 104.71 | 31.13 | 2 | Breeding line |
| AWASH1 | 152.50 | 15.67 | 164.00 | 87.00 | 104.79 | 19.60 | 3 | Breeding line |
| BAT477N | 179.50 | 48.33 | 162.00 | 30.00 | 104.96 | 19.50 | 1 | Breeding line |
| SMR127 | 81.50 | 78.33 | 68.00 | 193.00 | 105.21 | 25.18 | 3 | Breeding line |
| NABE17 | 38.50 | 140.33 | 141.00 | 101.00 | 105.21 | 30.31 | 2 | Released variety |
| MAB_373 | 165.00 | 42.67 | 85.00 | 131.00 | 105.92 | 23.45 | 1 | Breeding line |
| NUS5 | 34.00 | 131.67 | 143.00 | 119.00 | 106.92 | 27.31 | 2 | Breeding line |
| SMN105 | 119.00 | 83.67 | 168.00 | 57.00 | 106.92 | 21.91 | 1 | Breeding line |
| CAL96 | 79.50 | 170.67 | 146.00 | 32.00 | 107.04 | 34.38 | 2 | Breeding line |
| NUA702 | 152.50 | 141.67 | 96.00 | 38.00 | 107.04 | 25.53 | 1 | Breeding line |
| KFRR287 | 40.50 | 191.00 | 182.00 | 22.00 | 108.88 | 27.13 | 2 | Breeding line |
| SMC22 | 161.00 | 69.33 | 197.00 | 10.00 | 109.33 | 23.97 | 1 | Breeding line |
| MAB_354 | 155.50 | 91.33 | 150.00 | 41.00 | 109.46 | 24.24 | 1 | Breeding line |
| NABE4 | 116.00 | 171.00 | 44.00 | 107.00 | 109.5 | 35.90 | 2 | Released variety |
| SMN115 | 124.00 | 54.00 | 121.00 | 142.00 | 110.25 | 24.56 | 3 | Breeding line |
| NAROBEAN3 | 25.50 | 184.67 | 186.00 | 46.00 | 110.54 | 27.36 | 2 | Released variety |
| KARP22 | 149.00 | 80.00 | 133.00 | 81.00 | 110.75 | 20.11 | 1 | Breeding line |
| NUS16 | 122.00 | 126.67 | 120.00 | 77.00 | 111.42 | 31.78 | 2 | Breeding line |
| NABE14 | 75.00 | 185.33 | 165.00 | 23.00 | 112.08 | 32.41 | 2 | Released variety |
| SMC163 | 144.50 | 132.67 | 173.00 | 3.00 | 113.29 | 24.22 | 1 | Breeding line |
| KCN64 | 183.50 | 76.33 | 193.00 | 1.00 | 113.46 | 17.07 | 1 | Breeding line |
| UGK39 | 148.50 | 106.33 | 147.00 | 54.00 | 113.96 | 27.52 | 1 | Breeding line |
| ETHOPIA1 | 13.50 | 141.33 | 156.00 | 147.00 | 114.46 | 31.96 | 2 | Released variety |
| BFS88 | 154.00 | 64.33 | 131.00 | 109.00 | 114.58 | 25.83 | 1 | Breeding line |
| DAB919 | 10.50 | 177.00 | 100.00 | 173.00 | 115.13 | 26.10 | 2 | Breeding line |
| SMC16 | 164.00 | 92.00 | 194.00 | 11.00 | 115.25 | 25.77 | 1 | Breeding line |
| BFS113 | 176.50 | 94.33 | 53.00 | 139.00 | 115.71 | 22.00 | 1 | Breeding line |
| KCN12 | 161.50 | 117.00 | 74.00 | 112.00 | 116.13 | 22.78 | 1 | Breeding line |
| KCN10 | 191.00 | 49.33 | 199.00 | 28.00 | 116.83 | 20.52 | 1 | Breeding line |
| SMR197 | 105.00 | 64.00 | 154.00 | 146.00 | 117.25 | 22.01 | 1 | Breeding line |
| VAX1 | 168.00 | 64.33 | 89.00 | 148.00 | 117.33 | 20.30 | 1 | Breeding line |
| DAB91 | 39.00 | 155.00 | 166.00 | 110.00 | 117.5 | 28.98 | 2 | Breeding line |
| NUA668 | 119.00 | 131.00 | 177.00 | 43.00 | 117.5 | 23.18 | 1 | Breeding line |
| KND86 | 169.00 | 67.00 | 57.00 | 178.00 | 117.75 | 25.54 | 3 | Breeding line |
| KFRR39 | 90.00 | 157.33 | 189.00 | 35.00 | 117.83 | 27.35 | 2 | Breeding line |
| ACC31 | 111.50 | 131.67 | 64.00 | 165.00 | 118.04 | 37.97 | 2 | Breeding line |
| SMC201 | 155.00 | 92.67 | 170.00 | 56.00 | 118.42 | 24.14 | 1 | Breeding line |
| SMC24 | 150.50 | 104.67 | 190.00 | 29.00 | 118.54 | 23.18 | 1 | Breeding line |
| DAB249 | 38.00 | 148.67 | 128.00 | 161.00 | 118.92 | 29.81 | 2 | Breeding line |
| SMR245 | 123.00 | 84.00 | 117.00 | 154.00 | 119.5 | 24.42 | 1 | Breeding line |
| NABE22 | 71.00 | 193.00 | 192.00 | 31.00 | 121.75 | 25.03 | 2 | Released variety |
| BFS99 | 162.50 | 88.67 | 144.00 | 92.00 | 121.79 | 22.04 | 1 | Breeding line |
| BFS87 | 135.50 | 58.33 | 108.00 | 186.00 | 121.96 | 25.82 | 3 | Breeding line |
| SMR122 | 181.50 | 26.33 | 159.00 | 121.00 | 121.96 | 21.29 | 3 | Breeding line |
| NABE20 | 108.00 | 141.00 | 88.00 | 152.00 | 122.25 | 33.38 | 2 | Released variety |
| KCN9 | 187.50 | 26.33 | 127.00 | 150.00 | 122.71 | 20.91 | 1 | Breeding line |
| NUA671 | 113.00 | 151.00 | 157.00 | 72.00 | 123.25 | 24.08 | 1 | Breeding line |
| SMR128 | 96.50 | 98.67 | 101.00 | 197.00 | 123.29 | 22.84 | 3 | Breeding line |
| DAA332 | 11.00 | 180.33 | 111.00 | 192.00 | 123.58 | 36.41 | 2 | Breeding line |
| NABE21 | 36.00 | 194.33 | 171.00 | 97.00 | 124.58 | 30.68 | 2 | Released variety |
| NUS27 | 63.00 | 161.33 | 178.00 | 98.00 | 125.08 | 29.54 | 2 | Breeding line |
| JESCA | 13.50 | 169.33 | 169.00 | 149.00 | 125.21 | 25.88 | 2 | Breeding line |
| DAA129 | 24.00 | 176.00 | 180.00 | 123.00 | 125.75 | 34.44 | 2 | Breeding line |
| BFS142 | 111.50 | 65.00 | 153.00 | 177.00 | 126.63 | 24.69 | 1 | Breeding line |
| NE34-12-42 | 116.00 | 59.67 | 151.00 | 180.00 | 126.67 | 20.30 | 3 | Breeding line |
| SMR130 | 145.00 | 90.00 | 183.00 | 89.00 | 126.75 | 18.10 | 1 | Breeding line |
| MAB_349 | 112.50 | 66.00 | 163.00 | 169.00 | 127.63 | 20.55 | 1 | Breeding line |
| GGB2 | 145.50 | 49.33 | 137.00 | 179.00 | 127.71 | 25.04 | 3 | Breeding line |
| BFS137 | 162.00 | 33.33 | 148.00 | 168.00 | 127.83 | 22.63 | 3 | Breeding line |
| SMC141 | 176.00 | 123.33 | 119.00 | 94.00 | 128.08 | 27.00 | 1 | Breeding line |
| AAB_007 | 194.00 | 72.33 | 132.00 | 120.00 | 129.58 | 22.37 | 1 | Breeding line |
| DAA6 | 111.50 | 176.67 | 172.00 | 66.00 | 131.54 | 32.67 | 2 | Breeding line |
| NUA595 | 103.00 | 194.33 | 149.00 | 85.00 | 132.83 | 36.33 | 2 | Breeding line |
| DAB216 | 70.00 | 184.67 | 198.00 | 79.00 | 132.92 | 31.83 | 2 | Breeding line |
| SMC207 | 133.50 | 95.67 | 187.00 | 117.00 | 133.29 | 27.57 | 1 | Breeding line |
| NUA704 | 169.50 | 75.67 | 155.00 | 134.00 | 133.54 | 26.45 | 3 | Breeding line |
| KCN62 | 192.50 | 12.00 | 175.00 | 158.00 | 134.38 | 19.95 | 3 | Breeding line |
| BFS86 | 172.50 | 72.67 | 185.00 | 108.00 | 134.54 | 23.78 | 3 | Breeding line |
| BFS117 | 147.00 | 88.67 | 125.00 | 189.00 | 137.42 | 23.82 | 3 | Breeding line |
| G10677 | 150.00 | 93.33 | 130.00 | 182.00 | 138.83 | 20.70 | 3 | Breeding line |
| NUA711 | 108.00 | 148.67 | 109.00 | 190.00 | 138.92 | 31.43 | 2 | Breeding line |
| UGK85 | 113.00 | 134.00 | 142.00 | 170.00 | 139.75 | 30.45 | 2 | Breeding line |
| DAB13 | 102.5 | 98.67 | 195.00 | 163.00 | 139.79 | 20.89 | 1 | Breeding line |
| SEQ1027 | 199.00 | 178.67 | 181.00 | 4.00 | 140.67 | 31.36 | 1 | Breeding line |
| KFRR138 | 150.00 | 161.00 | 191.00 | 61.00 | 140.75 | 25.51 | 1 | Breeding line |
| SMR133 | 189.00 | 59.00 | 152.00 | 167.00 | 141.75 | 22.61 | 1 | Breeding line |
| BFS119 | 171.50 | 131.67 | 176.00 | 91.00 | 142.54 | 22.44 | 1 | Breeding line |
| NUA682 | 161.50 | 176.33 | 103.00 | 143.00 | 145.96 | 33.12 | 2 | Breeding line |
| SAB618 | 50.00 | 187.00 | 158.00 | 191.00 | 146.5 | 35.81 | 2 | Breeding line |
| NUA608 | 160.50 | 182.00 | 174.00 | 70.00 | 146.63 | 29.78 | 2 | Breeding line |
| NUA607 | 138.50 | 134.33 | 196.00 | 118.00 | 146.71 | 24.94 | 1 | Breeding line |
| KNG17-24 | 142.00 | 152.67 | 179.00 | 114.00 | 146.92 | 27.11 | 1 | Breeding line |
| MIB465 | 159.00 | 67.33 | 167.00 | 195.00 | 147.08 | 20.68 | 3 | Breeding line |
| SMR117 | 193.50 | 118.67 | 139.00 | 145.00 | 149.04 | 24.34 | 1 | Breeding line |
| NUS21 | 84.50 | 195.33 | 135.00 | 188.00 | 150.71 | 29.11 | 2 | Breeding line |
| SMC168 | 189.00 | 111.00 | 145.00 | 164.00 | 152.25 | 22.54 | 1 | Breeding line |
| SMR221 | 173.50 | 101.67 | 138.00 | 198.00 | 152.79 | 24.19 | 1 | Breeding line |
| ADP-58 | 190.00 | 135.33 | 161.00 | 184.00 | 167.58 | 21.04 | 1 | Breeding line |
| CMKFS105 | 193.00 | 134.00 | 188.00 | 175.00 | 172.5 | 26.14 | 1 | Breeding line |
